# Current Progress in the Structural and Biochemical Characterization of Proteins Involved in the Assembly of Lipopolysaccharide

**DOI:** 10.1155/2018/5319146

**Published:** 2018-11-25

**Authors:** Heather O. Bohl, Hideki Aihara

**Affiliations:** Department of Biochemistry, Molecular Biology, & Biophysics, University of Minnesota, 321 Church Street, 6-155 Jackson Hall, Minneapolis, MN 55455, USA

## Abstract

The lipid component of the outer leaflet of the outer membrane of Gram-negative bacteria is primarily composed of the glycolipid lipopolysaccharide (LPS), which serves to form a protective barrier against hydrophobic toxins and many antibiotics. LPS is comprised of three regions: the lipid A membrane anchor, the nonrepeating core oligosaccharide, and the repeating O-antigen polysaccharide. The lipid A portion is also referred to as endotoxin as its overstimulation of the toll-like receptor 4 during systemic infection precipitates potentially fatal septic shock. Because of the importance of LPS for the viability and virulence of human pathogens, understanding how LPS is synthesized and transported to the outer leaflet of the outer membrane is important for developing novel antibiotics to combat resistant Gram-negative strains. The following review describes the current state of our understanding of the proteins responsible for the synthesis and transport of LPS with an emphasis on the contribution of protein structures to our understanding of their functions. Because the lipid A portion of LPS is relatively well conserved, a detailed description of the biosynthetic enzymes in the Raetz pathway of lipid A synthesis is provided. Conversely, less well-conserved biosynthetic enzymes later in LPS synthesis are described primarily to demonstrate conserved principles of LPS synthesis. Finally, the conserved LPS transport systems are described in detail.

## 1. Introduction

**Figure 1:**
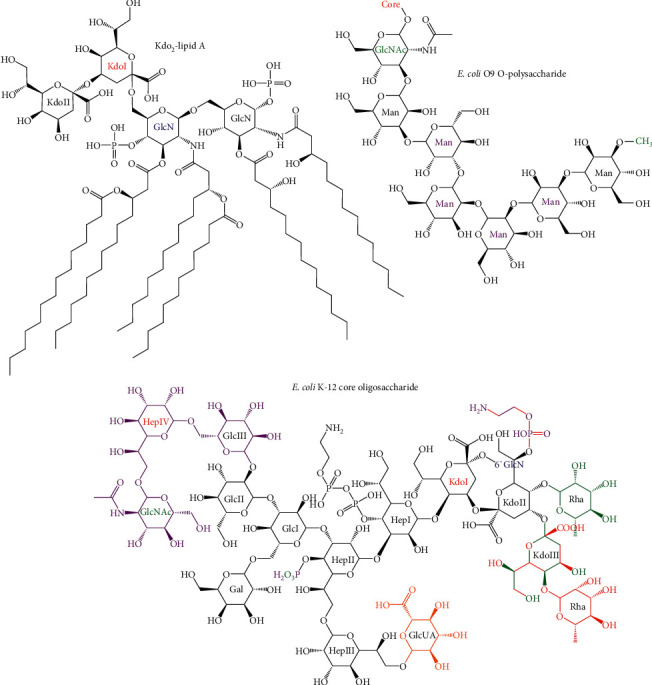
LPS chemical structure. Chemical structures of the canonical lipid A of *E. coli*, the core oligosaccharide of *E. coli* K-12, and the *E. coli* O9 O-antigen (showing one repeat unit) [1]. The text for the distal glucosamine of lipid A attached to the core oligosaccharide is colored blue. The text for the residues at each end of the core oligosaccharide is colored red. The text for the ends of the O-antigen is colored green, and that for the repeat region is colored purple. In the core oligosaccharide, atom and bond coloring indicates different core structures [3]. Black indicates the conserved structure, and purple, red, and green indicate moieties present in three different variants of the K-12 core. Glucuronic acid (GlcUA, orange) may be attached to HepIII in the absence of the phosphate on HepII [4]. Dual coloring of moieties indicates their presence in both variants. Chemical structure figures were generated with ChemBioDraw Ultra 14.0.

Gram-negative bacteria are distinguished from Gram-positive bacteria by the secondary lipid bilayer that surrounds their peptidoglycan cell wall [1]. In the majority of Gram-negative bacteria, this outer membrane is an asymmetric bilayer with phospholipids on the inner leaflet and lipopolysaccharide (LPS) on the outer leaflet [1, 2]. LPS is a glycolipid composed of a lipid A membrane anchor, a core oligosaccharide, and a repeating O antigen polysaccharide (Figure 1) [1, 5]. This layer of LPS provides a permeability barrier to environmental toxins. When this layer of LPS is disrupted by invasion of phospholipids [2], loss of LPS synthesis or transport [6], or loss of glycosylation [7], the bacteria show increased sensitivity to hydrophobic toxins, such as detergent or bile salts or antibiotics. Furthermore, a complete loss of LPS by mutation of lipid A synthesis genes made bacteria less virulent to *Caenorhabditis elegans* and human epithelial cells [8].

The role of LPS in disease is related not only to its protective function in Gram-negative bacteria but also to its interaction with the host's innate immune system. Humans possess a cohort of proteins that allow rapid response to the presence of LPS [9]. LPS binding protein is thought to extract LPS from bacterial membranes and present an LPS molecule to CD14 [9, 10]. CD14, which may be secreted or lipid-linked to the extracellular face of cells, transfers this LPS to the complex of myeloid differentiation factor 2 and toll-like receptor 4 (MD2/TLR4) [9, 11–13]. Activation of TLR4 by LPS leads to the production and secretion of proinflammatory cytokines and type I interferon [9, 14]. While this response is beneficial for clearing small bacterial infections, overstimulation of the inflammatory response during systemic infection, such as sepsis (presence of bacteria in the blood), is potentially fatal and can cause permanent organ damage or neurological problems [15, 16].

Because of the severity of this condition, rapid treatment with broad-spectrum antibiotics before the causative bacteria can be identified is recommended for patients with sepsis [15]. The highly conserved and (in most cases) essential enzymes involved in the synthesis of the lipid A portion of LPS are promising targets for the development of new broad-spectrum antibiotics to treat sepsis and infections by antimicrobial-resistant Gram-negative strains [1, 7, 17, 18].

## 2. Main Text

### 2.1. Raetz Pathway

**Figure 2:**
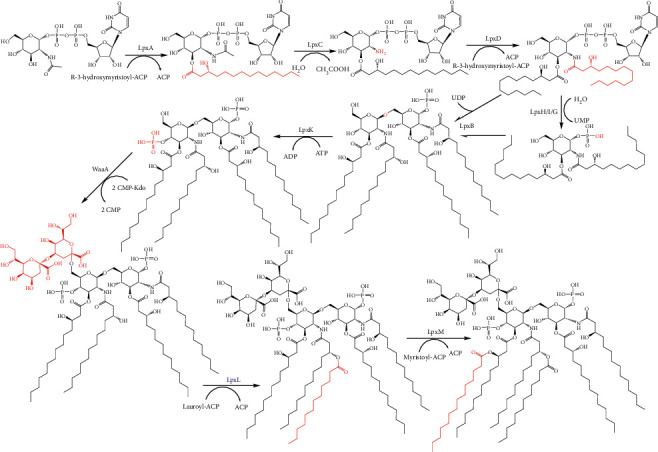
Raetz pathway of lipid A synthesis. The moieties added in each step are shown in red [1].

The canonical Raetz pathway of lipid A synthesis is a 9 enzyme pathway that produces Kdo_2_-lipid A (Figure 2) [1]. While some variation exists between species, particularly with regards to acylation, the Raetz pathway, especially the 4 of the first 5 enzymes, is well conserved in Gram-negative bacteria, excluding specialized species that do not produce LPS [17, 19]. Most of these enzymes have been characterized at the biochemical and structural levels and the following summarizes what is known about the enzymes of the Raetz pathway.

#### 2.1.1. LpxA

Early experiments with radiolabeled substrates indicated that UDP-*N*-acetylglucosamine is incorporated into the lipid A synthesis pathway and suggested that acylation of the 3-hydroxyl of glucosamine precedes the removal of acetate [20]. The acyltransferase responsible for the addition of this acyl chain was denoted LpxA and was found to be specific for UDP-*N*-acetylglucosamine and R-3-hydroxymyristoyl-acyl carrier protein (ACP), with little activity toward the S-enantiomer or the coenzyme A adduct of *β*-hydroxymyristate or toward palmitoyl-ACP, myristoyl-ACP, or acyl chain acceptors with chains more than 3 carbons long at the 2-amine of glucosamine or with uracil substituted with any base other than thymine [21, 22].

Furthermore, radiolabeled LpxA product was converted to other intermediates of lipid A synthesis [21].

**Figure 3:**
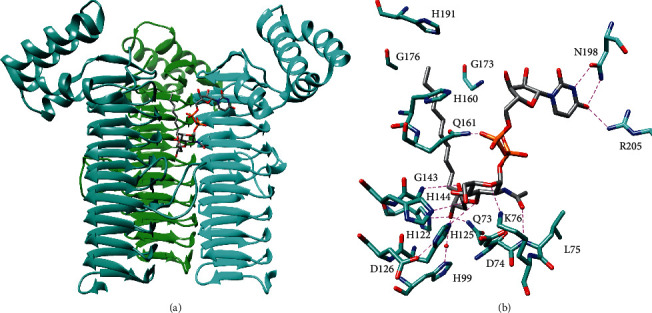
LpxA: (a) ribbon diagram of the LpxA trimer bound to its UDP-3-O-(R3-hydroxymyristoyl)-*N*-acetylglucosamine product (PDB: 2QIA) [23]; (b) residues involved in product binding from (a). Subscripts are used below to distinguish subunits in the trimer. Hydrogen-bonding interactions are shown. Distances of interactions in Ångstroms are as follows: 3.3 from 3′-acyl carbonyl O to G143_0.3_ N 3.2 from uracil O4 to N198_0.1_ N*δ*, 3.0 from uracil O4 to R205_0.1_ N*η*2 2.7 from uracil N3 to N198_0.1_ O*δ*, 2.9 from water to H99_0.3_ N*δ*, 2.8 from same water to *β*-hydroxyl, 2.8 from *β*-hydroxyl to H122_0.3_ N*ε*, 3.0 from *β*-hydroxyl to Q73_0.3_ N*ε*, 3.3 from glucosamine 3′O to H125_0.3_, 2.8 from H125_0.3_ N*δ* to D126_0.3_, 2.9 from glucosamine 6′-hydroxyl to H144_0.3_ N*ε*, 3.0 from the glucosamine ring O to K76_0.3_ N*ζ*, 3.0 from 2′-acetyl carbonyl to L75_0.3_ N, and 2.6 from *α*-phosphate to Q161_0.3_ N*ε*. Protein structure figures were generated using UCSF Chimera [24].

Crystal structures of LpxA revealed that LpxA is composed of an unusual *N*-terminal left-handed parallel *β*-helix domain and a C-terminal *α*-helical domain and forms a trimer with 3-fold symmetry (Figure 3) [25]. Each *β*-helix approximates an equilateral triangular prism, and these helices come together in the trimer to form a large cleft between each pair of subunits [25]. Extended loops within helical repeats 4 and 5 form additional contacts between adjacent subunits [26]. Crystal structures with UDP-*N*-acetylglucosamine or the product bound show that substrates bind in this cleft between subunits with the uridine and *N*-acetylglucosamine moieties contacting adjacent subunits and with the R-3-hydroxymyristoyl chain extending up the cleft toward the C-terminal domain (Figure 3) [23, 26]. The extended loop within helical repeat 4 provides part of the contacts for *N*-acetylglucosamine [23, 26].

Furthermore, these crystal structures provide insights into the mechanism and selectivity of LpxA [23, 26]. LpxA is thought to catalyze the nucleophilic attack of the glucosamine 3-hydroxyl on the thioester of R-3-hydroxymyristoyl-ACP, and a conserved His (H125 in *E. coli* LpxA) is positioned to act as the catalytic base and accept the proton from this hydroxyl, while the backbone amide of G143 is positioned to stabilize the oxyanion intermediate [23, 26]. The role of H125 was supported by activity assays that showed it to be critical for acyl transfer [27]. D126 hydrogen bonds with and stabilizes the position of H125 forming a catalytic dyad similar to that observed in other acyltransferases [26]. In addition, the product-bound structure suggests that H191 of *E. coli* LpxA limits the length of the acyl chain by capping the channel, wherein the chain binds and mutation of another residue that lines this channel (G173) to larger residues decreased activity toward the natural acyl donor substrate and increased activity toward the 10 carbon chain analogue [23, 28]. Moreover, selection of the R-3-hydroxylated fatty acid may be controlled by hydrogen-bonding interactions between this functional group and H122, Q73, and an ordered water bound to H99 [23]. Finally, UDP-*N*-acetylglucosamine selectivity is governed by extensive hydrogen-bonding as well as the stereospecific architecture of the *N*-acetylglucosamine binding pocket that would interfere with the binding of a sugar with axial substituents (Figure 3) [26].

#### 2.1.2. LpxC

LpxC catalyzes the second step in the Raetz pathway, the deacetylation of UDP-3-O-(R3-hydroxymyristoyl)-*N*-acetylglucosamine [29, 30]. Early experiments showed that the LpxA product is deacetylated when incubated with *E. coli* extracts and that the deacetylated product can be converted into other intermediates in the pathway [29]. Furthermore, LpxC was found to have poor activity toward UDP-*N*-acetylglucosamine [31]. Because the reaction catalyzed by LpxA is freely reversible and, surprisingly, thermodynamically unfavorable, LpxC catalyzes the committed step of lipid A synthesis [22]. In accordance with the thermodynamic importance of this step, LpxC is also an important point of regulation for lipid A synthesis [32, 33]. LpxC concentration is regulated by proteolytic degradation by FtsH, which may be controlled by the levels of acyl-ACP or Raetz pathway intermediates [32, 33]. In addition, the heat shock-induced protein LapB was found to be essential for this function of FtsH [34]. LapB likely acts as a scaffold or chaperone for inner membrane proteins involved in LPS synthesis and transport including FtsH, LpxM, and WaaC [34].

LpxC is a Zn^2+^-dependent metalloenzyme though it displays higher activity in the presence of Ni^2+^ or Co^2+^, which may be due to inhibition by Zn^2+^ binding at a second site [31]. Consistent with these enzymatic assays, the crystal structure of *Aquifex aeolicus* LpxC was solved with two Zn^2+^ in the active site, representing the zinc-inhibited state, and the crystal structure of *E. coli* LpxC in complex with the lipidic product, which was crystallized in a high concentration of phosphate, was solved with a single high-affinity Zn^2+^ in the active site [35, 36]. Native mass spectrometry was also consistent with the binding of one high-affinity Zn^2+^ [36].

**Figure 4:**
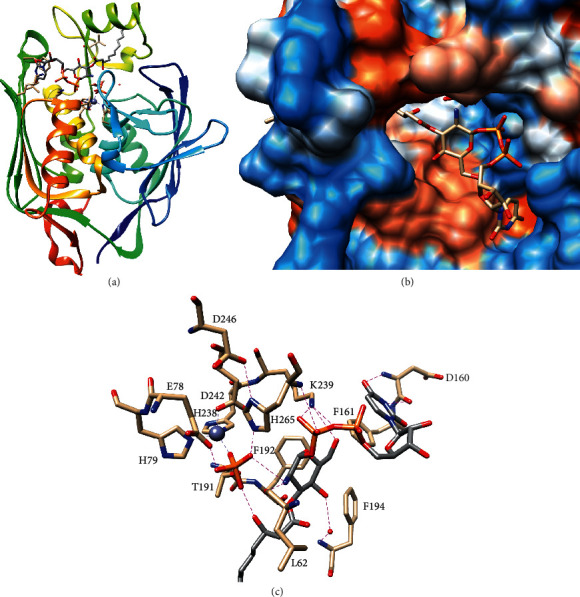
LpxC. (a) Ribbon structure of LpxC bound to its UDP-3-O-(R3-hydroxymyristoyl)-glucosamine product (PDB: 4MDT) [36]. Spectrum coloring begins with blue at the *N*-terminus. (b) Hydrophobicity surface of the structure in (a) with a orange-blue scale. Orange is more hydrophobic. (c) Residues involved in product binding from (a). Zinc coordination is shown in purple, and hydrogen-bond and salt-bridge interactions are shown in pink. Distances of interactions in Ångstroms are as follows: 1.9 from Zn^2+^ to H238 N*ε*, 2.0 from Zn^2+^ to H79 N*ε*, 2.0 from Zn^2+^ to D242 O*δ*1, 1.9 from Zn^2+^ to phosphate O2, 2.5 from water to *β*-phosphate and 3.2 to *α*-phosphate and 3.0 to A266 N 3.2 from pyrophosphate bridging O and 2.8 from *β*-phosphate and 3.5 from glucosamine 6′-hydroxyl to K239 N*ζ*, 2.8 from *β*-hydroxyl to phosphate O1, 3.2 from water to F194 N and 2.9 to glucosamine 4′-hydroxyl, 3.1 from glucosamine 2′-amine to phosphate O3 and 2.7 to L62 O 2.6 from E78 O*ε*2 to phosphate O4, 2.4 from H265 N*ε* to phosphate O3, 3.5 from uracil O4 to D160 N 2.7 from D246 O*δ*2 to H265 N*δ*, and 3.5 from uracil N3 to D160 O.

Solution NMR and crystal structures of LpxC revealed that the enzyme is composed of two *α*/*β*-domains with a conserved topology that form a five-stranded *β*-sheet and two *α*-helices [35, 37]. The *α*-helices of the conserved fold are packed together giving LpxC an overall pseudo-twofold symmetry (Figure 4) [35, 37]. The domains contain divergent insertions between the fourth *β*-strand and first *α*-helix: the *N*-terminal domain's insertion forms a three-stranded *β*-sheet, and the C-terminal domain's insertion contributes a short helix to the helical core and forms an *α*/*β*-subdomain [35–37].

The structure of *E. coli* LpxC with UDP-3-O-(R-3-hydroxymyristoyl)-glucosamine and phosphate bound in the active site shows that the product primarily binds to the insertions and the C-terminal domain with the first helix of the *N*-terminal domain contributing one Zn^2+^-coordinating residue and the putative catalytic base [36]. Zn^2+^ is coordinated with tetrahedral geometry by two His side chains: one Asp side chain (H79, H238, and D242 in *E. coli* LpxC) and a molecule from the crystallization solution [35–37]. The importance of these residues was supported by enzymatic assays that showed decreased activity in mutants of any one of these residues as well as greatly decreased Zn^2+^-binding in the His to Ala mutants [38].

In the product-bound structure of *E. coli* LpxC, the fourth ligand of Zn^2+^ was modeled as phosphate, and it may approximate the tetrahedral transition state of the leaving group and hydrogen bonding with the glucosamine amine [36]. The phosphate forms hydrogen bonds with E78, T191, and H265 implicating these residues in catalysis, and mutants of these residues were indeed found to have decreased activity [36, 38, 39]. Specifically, E78 is positioned to accept a proton from a Zn^2+^-coordinated hydrolytic water, and this role was supported by the greater reduction in activity of E78Q in comparison to E78 A as E78Q cannot act as a catalytic base but likely blocks the compensatory positioning of another residue or solvent molecule [36, 38]. On the contrary, H265 is positioned to donate a proton to the glucosamine amine, and D246, which hydrogen-bonds with the opposite nitrogen of the H265 azole thus tuning pK_a_ of H265, was also critical for activity [36, 38].

As stated above, the product primarily interacts with the insertions and the C-terminal domain [36]. The UDP moiety binds to the C-terminal domain: uracil hydrogen-bonds with the backbone of D160 and forms a π-stacking interaction with F161, and the pyrophosphate interacts with K239 and the backbone of H265 (Figure 4) [36]. Recognition of the glucosamine moiety involves all three subdomains [36]. The 6′-hydroxyl also hydrogen-bonds with K239, and the 2′-amine hydrogen-bonds with the backbone of L62 (Figure 4). The importance of K239 was confirmed by enzymatic assays showing a slightly increased *K*_M_ and greatly decreased *k*_cat_ of the K239 A mutant [39]. In addition, the 4′-hydroxyl hydrogen-bonds to an ordered water bound to the F194 backbone [36]. Finally, the acyl chain binds in a hydrophobic channel formed by the C-terminal domain insertion with the chain terminus extending into solvent, suggesting that, while presence of the chain is critical for activity, LpxC has little specificity for chain length (Figure 4) [31, 35, 36].

#### 2.1.3. LpxD

Early experiments indicated that addition of the second R3-hydroxymyristoyl group at the glucosamine 2-amino group precedes the hydrolysis of the phosphoanhydride of UDP [20]. This structural gene for the UDP-3-O-(R3-hydroxymyristoyl)-glucosamine-*N*-acyltransferase (*lpxD*) was identified by homology to *lpxA* and its position in the same operon, and enzymatic assays showed LpxD is highly specific for *β*-hydroxymyristoyl-ACP, showing no activity for myristoyl-ACP and 10-fold reduced activity for *β*-hydroxylauroyl-ACP [40, 41]. Furthermore, steady-state kinetic experiments showed that LpxD follows an ordered sequential mechanism, wherein R3-hydroxymyristoyl-ACP binds first and ACP dissociates last [41].

Crystal structures of LpxD reveal many similar features shared with LpxA [42, 43]. In particular, LpxD has a very similar left-handed *β*-helix domain that includes two elongated loops (within helical repeats 5 and 6) that contribute additional interactions with the adjacent subunit in the trimer [42]. Moreover, LpxD also has an *α*-helical C-terminal domain, although in LpxD, the C-terminal helices of the subunits come together to form a trimeric helix bundle [42].

**Figure 5:**
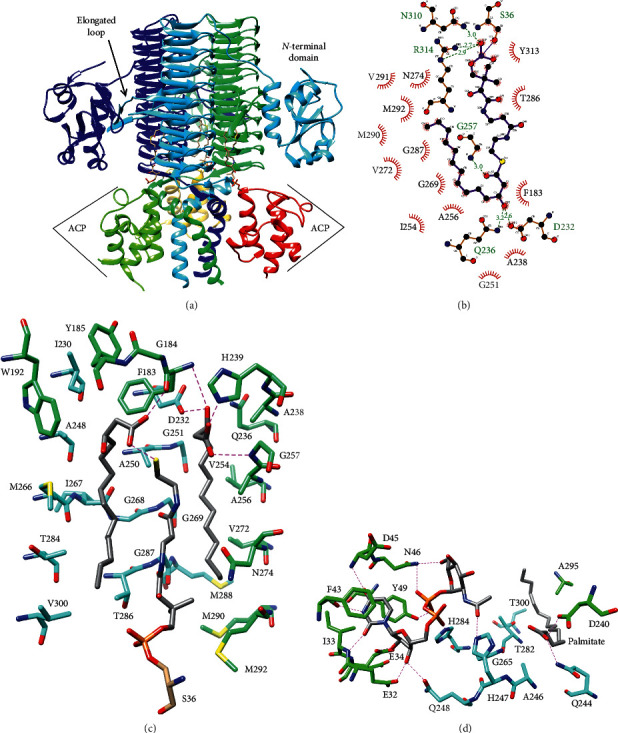
LpxD. (a) Heterohexamer of LpxD and R3-hydroxymyristoyl-ACP (PDB: 4IHF) [44]. ACP chains are colored red, yellow, and chartreuse, and LpxD chains are colored blue, cyan, and sea green. (b) LigPlot+ diagram of acyl donor substrate from (a) [45]. Distances of hydrogen-bond and salt-bridge interactions shown in Ångstroms are as follows: 3.2 from acyl chain *β*-hydroxyl to Q236 N*ε* and 2.6 to D232 O*δ*2, 3.0 from acyl chain carbonyl to G257 N 3.0 from phosphate to N310 N*δ* and 2.7 and 2.9, respectively, to R314 N*η* and N*ε*. (c) Acyl chain binding in LpxD/ACP heterohexamer (PDB: 4IHG) [44]. Subscripts are used below to distinguish protein chains in the complex. Hydrogen-bonds are shown, and corresponding distances in Ångstroms are as follows: 2.9 from putative 2′-R3-hydroxymyristate carboxylate to G257_C_ N 2.1 from putative 2′-R3-hydroxymyristate carboxylate to H239_C_ N*ε*, 3.0 from putative 2′-R3-hydroxymyristate *β*-hydroxyl to D232_B_ O*δ*2 and 3.6 to F183_C_ N and 2.8 from putative 3′-R3-hydroxymyristate carboxylate to F183_C_ O and 3.2 to S of phosphopantetheine attached to ACP S36_H_. (d) UDP-*N*-acetylglucosamine and palmitate binding to LpxD trimer (PDB: 2IU9) [42]. Hydrogen-bonds are shown with the following distances in Ångstroms: 2.8 from palmitate carboxylate to Q244_A_ N*ε* and 2.9 to D240_B_ O*δ*2, 2.6 from 2′-acetyl carbonyl to H247_A_ N*ε*, 2.8 from uracil O2 to I33_B_ N 2.7 from uracil N3 to F43_B_ O 2.8 from uracil O4 to D45_B_ N 2.6 from ribose 3′-hydroxyl to E32_B_ O*ε*2 and 2.9 to Q248_A_ O*ε*, 3.0 from ribose 2′-hydroxyl to E34_B_ O*ε*1, 2.4 from *β*-phosphate to Y49_B_ O*η* and 3.2 to N46_B_ N*δ*, 2.7 from *α*-phosphate to H284_A_ N*ε*, and 3.1 from glucosamine 6′-hydroxyl to N46_B_ N*δ*.

However, LpxD has an additional *N*-terminal domain composed of a five-stranded *β*-sheet surrounded by *α*-helices and a two-stranded *β*-sheet (Figure 5) [42]. Furthermore, crystal structures of LpxD bound to substrates and substrate analogues have revealed conserved catalytic residues and mechanisms of substrate specificity [42, 44]. Specifically, a conserved His (H239 in *E. coli*) is positioned to accept a proton from the glucosamine amine, while the G257 amide is positioned to stabilize the oxyanion intermediate [42–44]. As in LpxA, the substrates bind at the cleft between subunits [42, 44].

In the structure of *Chlamydia trachomatis* LpxD, UDP-*N*-acetylglucosamine binds to the *N*-terminal domain and the left-handed *β*-helix domain of the adjacent subunit with the *N*-terminal domain primarily binding the uridine moiety [42]. At the active site formed by chains A and B, uracil is sandwiched in *π*-stacking interactions with F43_B_ and Y49_B_ (F41 and Y47 in *E. coli*) and forms hydrogen-bonds with the backbone of residues 33_B_ and 43_B_, and 45_B_ (Figure 5) [42]. Ribose is hydrogen-bonded to E32_B_ and E34_B_ (S30 and Q32 in *E. coli*) and Q248_A_ (Q240 in *E. coli*), while the pyrophosphate is engaged by Y49_B_, N46_B_, and H284_A_ (N44 and H276 in *E. coli*), and *N*-acetylglucosamine binds to N46_B_ and H247_A_ (H239 in *E. coli*, the predicted catalytic base) [42]. Kinetic assays in *E. coli* LpxD confirmed that H239 is critical for catalysis: H239 A decreased *k*_cat_ almost 3 orders of magnitude with a moderate decrease of the acyl acceptor *K*_M_ [41]. In addition, F41 was confirmed to be critical for acyl acceptor binding: F41 A increased the acceptor *K*_M_ 29-fold with only a small decrease in *k*_cat_ [41]. Conversely, Y47 A had little effect, and H276 A decreased *k*_cat_ 32-fold [41].

*E. coli* LpxD crystallized in complex with ACP yielded structures with R3-hydroxymyristoyl-ACP and with holo-ACP and two *β*-hydroxymyristate molecules bound to LpxD [44]. ACP primarily binds to the C-terminal domain with the pantetheine arm on ACP S36 extending up the active site cleft 0(Figure 5) [44]. Association with ACP is largely electrostatic with an acidic patch on ACP contacting a basic patch on LpxD [44]. For example, the Ala mutant of R193, which forms an ion pair with E41 of ACP, was found to cause a 23-fold increase of the R3-hydroxymyristoyl-ACP *K*_M_ [41, 44]. In the intact substrate structure, the R3-hydroxymyristoyl chain turns 180° and extends back toward the C-terminus of LpxD deeper in the cleft [44]. Specificity for the length of the acyl chain attached to ACP is conferred by the length of the hydrophobic pocket that accommodates the chain [41, 42, 44]. In *E. coli* LpxD, the ∼13 Å deep pocket is terminated by M290, and M290 A allowed incorporation of 16 and 18 carbon acyl chains *in vivo* [43]. Moreover, in *C. trachomatis* LpxD, which transfers a *β*-hydroxyarachidoyl chain, the corresponding residue is G298, and the hydrophobic pocket is ∼18 Å deep [42]. In the active site formed by chains B and C, the *β*-hydroxyl group of the transferred chain is recognized by hydrogen-bonds to D232_B_ and Q236_C_ of *E. coli* LpxD, and the acyl chain carboxylate hydrogen-bonds to the G257_C_ amide, consistent with its proposed stabilization of the oxyanion (Figure 5) [41, 43, 44]. Finally, the structure of *E. coli* LpxD bound to holo-ACP and two *β*-hydroxymyristates reveals the binding pocket for the 3′-acyl chain of the acceptor substrate: this pocket is formed by the left-handed *β*-helix of the subunit that contributes the *N*-terminal domain, the elongated loop of repeat 5 from the adjacent subunit, and the pantetheine group (Figure 5), consistent with kinetic experiments that showed R3-hydroxymyristoyl-ACP binds first [41, 44].

#### 2.1.4. LpxH

Early experiments indicated that hydrolysis of the phosphoanhydride of UDP-2,3-bis[O-(3R)-3-hydroxymyristoyl]-glucosamine (UDP-DAG) is the fourth step in the Raetz pathway [20, 46]. The responsible pyrophosphatase in *E. coli* (LpxH) was identified as an essential Mn^2+^-dependent hydrolase that catalyzes the attack of water on the *α*-phosphate of UDP-DAG [47–49]. Electron paramagnetic resonance was utilized to further characterize Mn^2+^ binding, and these data were consistent with the presence of a binuclear manganese center [49].

**Figure 6:**
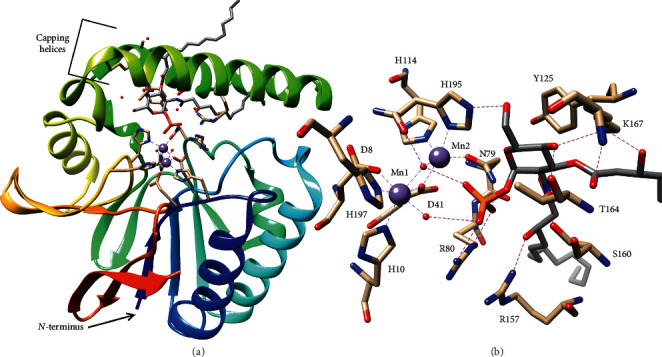
Pseudomonas LpxH bound to the lipid X product. (a) Ribbon structure of PaLpxH bound to lipid X (PDB: 5B49) [50]. Lipid X carbons are colored grey, and protein carbons are colored tan. Mn^2+^ ions are colored purple. Spectrum coloring begins with blue at the *N*-terminus. (b) Residues involved in lipid X binding from (a). Metal coordination is shown in purple with the following distances in Ångstroms: from Mn301 to H10 N*ε* (2.3), to H197 N*ε* (2.3), to D8 O*δ*1 (2.2), to D41 O*δ*2 (2.3), to bridging water (2.3), and to adjacent water (2.2) and from Mn302 to D41 O*δ*2 (2.2), to N79 O*δ* (2.1), to H195 N*δ* (2.3), to H114 N*ε* (2.2), and to bridging water (2.2). Hydrogen-bond and salt-bridge interactions are shown in pink with distances in Ångstroms of 2.7 from H195 O to bridging water, 2.9 from 2-acyl chain *β*-hydroxyl to R157 N*η*1, 2.8 from glucosamine 4-hydroxyl to T164 O*γ* and 2.9 to K167 N*ζ*, 3.3 from K167 N*ζ* to 3-acyl chain carbonyl and 2.8 to its *β*-hydroxyl, 2.8 from glucosamine 6-hydroxyl to H195 N*ε*, 2.8 from glucosamine 2-amino to S160 O*γ*, and 2.7 from 1-phosphate to bridging water and 3.4 to adjacent water and 2.6 to N79 N*δ* and 2.8 and 3.1, respectively, to R80 N*ε* and N*η*2.

Crystal structures of LpxH from *Haemophilus influenzae* and *Pseudomonas aeruginosa* revealed that LpxH has a calcineurin-like metal-dependent phosphoesterase fold with the addition of a unique helical cap, comprising 3 major *α*-helices, that covers the active site and binds the lipid substrates (Figure 6) [50, 51]. The core domain is composed of 2 five-stranded *β*-sheets surrounded by 4 *α*-helices, and the helical cap domain is inserted between *β*-strand 6 and the final *α*-helix [50, 51]. The crystal structures confirm that LpxH has a binuclear manganese center in its active site [50, 51]. In the *P. aeruginosa* structure (PDB: 5B49), the first Mn^2+^ is coordinated by D8, H10, D41 (bridges the Mn^2+^), H197, a bridging water, and a second water that completes the octahedral geometry and likely represents the attacking water [50]. The second Mn^2+^ is coordinated by D41, the bridging water, N79, H114, and H195, which leaves one open coordination site that may be filled by the *α*-phosphate in the substrate-bound structure (Figure 6) [50]. The importance of several of the Mn^2+^-coordination residues was confirmed by enzymatic assays in *H. influenzae* LpxH (HiLpxH) that showed large (3–5 orders of magnitude) drops in specific activity when individual residues were mutated to Ala [49].

Structures of LpxH bound to its product, 2,3-bis[(3R)-3-hydroxymyristoyl]-glucosaminyl-1-phosphate (lipid X), show that the lipid X head group is recognized by extensive hydrogen-bonding interactions, and the acyl chains bind in the hydrophobic pocket formed by the capping domain and the interface with the core domain: the amide-linked chain is buried in the hydrophobic pocket while the ester-linked chain exits between the helices and extends across the top of the lid or into solvent [50, 51]. In the *P. aeruginosa* LpxH (PaLpxH) structure (PDB: 5B49), the lipid X phosphate is bound by N79 and R80, which is a conserved residue particular to LpxH and is usually His in other metal-dependent phosphoesterases of the calcineurin-like superfamily [50].

Consistent with the importance of this Arg, the Ala mutation in HiLpxH decreased activity 7000-fold [49]. In addition, glucosamine is hydrogen-bonded to S160, T164, K167, and H195, and the *β*-hydroxyl groups of the acyl chains are hydrogen-bonded to R157 and K167 (Figure 6) [50]. Finally, Y125 packs on top of the glucosamine ring [50]. All of these residues noted from PaLpxH are conserved in *E. coli* LpxH (same numbering) and HiLpxH (numbering +1) with the exception of T164, which is Asn and Lys, respectively.

**Figure 7:**
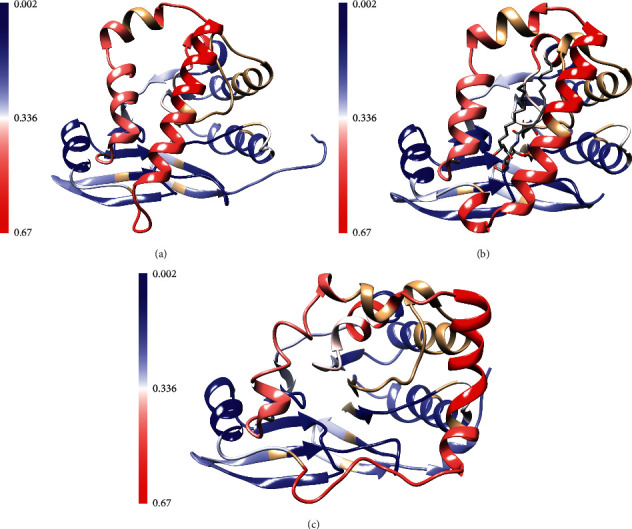
Structural dynamics of LpxH cap. PaLpxH ribbons are colored by fractional deuterium uptake at 10 min observed in hydrogen-deuterium exchange experiments of solubilized *E. coli* LpxH [52]. Blue indicates less deuterium uptake. (a) Closed/collapsed cap model observed in molecular dynamics simulations of PaLpxH-F82 G/L83 G [52]. (b) Lipid X-bound PaLpxH crystal structure (PDB: 5B49) [50]. (c). Open cap model observed in molecular dynamics simulations of PaLpxH without lipid X bound [52].

Furthermore, the structural dynamics of the substrate-binding cap domain have been explored by hydrogen-deuterium exchange and molecular dynamics simulations [52]. The relatively rapid hydrogen-deuterium exchange rate of the capping helices in a solubilized form of *E. coli* LpxH supported the idea that the capping domain is highly flexible in the absence of the substrate (Figure 7) [52]. Molecular dynamics simulations were consistent with the highly flexible nature of the capping domain in the absence of the substrate and predicted that capping helices of apo PaLpxH could undergo an opening motion, wherein the helices spread apart to expose the active site [52] (Figure 7(c)). In addition, molecular dynamics simulations predicted that a loop of the core pyrophosphatase containing F82 and L83 acts like a wedge to open the capping helices and that mutation of these residues to Gly would result in the collapse of the capping domain into a fully closed state (Figure 7(a)) [52]. Activity assays with solubilized *E. coli* LpxH confirmed that the F82 G/L83 G mutations significantly decreased activity [52].

#### 2.1.5. LpxG and LpxH2

*LpxG* was identified as a gene from *Chlamydia trachomatis* that could complement a *δlpxH* strain of *E. coli* [53]. Activity assays showed that, like LpxH, LpxG is a Mn^2+^-dependent pyrophosphatase that catalyzes the nucleophilic attack of water on the *α*-phosphate of UDP-DAG [49, 53]. Consistently, mutation of the predicted metal-coordinating residue D59 to Ala significantly decreased activity [53]. Also like LpxH, homology suggests that LpxG is a member of the calcineurin-like metal-dependent phosphoesterase family [53]. However, LpxG is predicted to have an additional *N*-terminal transmembrane helix, and the sequence identity with LpxH is very low (11%) [53]. Furthermore, while sequence alignments suggest that LpxG has a similarly located insertion in the calcineurin-like metal-dependent phosphoesterase fold, and the length and sequence of the insertions in LpxH and LpxG are different, suggesting that these proteins either diverged early in their evolution or arose by convergent evolution from different phylogenetic clades within the same enzyme family [50]. The structure of LpxG has not been determined.

Another gene called *lpxH2* because of its sequence homology to *lpxH* is present in some Gram-negative bacteria without *lpxH* but can also be present in addition to *lpxH* [17, 47]. Expression of LpxH2 could not complement the *δlpxH* strain of *E. coli*, and the sequence of the substrate-binding helical cap domain of LpxH is not conserved in LpxH2 [47, 50]. Thus, the function of *lpxH2* and its relevance to lipid A synthesis remains unknown.

#### 2.1.6. LpxI

LpxI is a nonhomologous alternative to LpxH present in some Gram-negative bacteria that lack LpxH and LpxG, particularly *α*-proteobacteria [17, 54]. *LpxI* was discovered as a gene of unknown function present in the same operon as *lpxA*, *lpxD*, and *lpxB* in bacteria lacking *lpxH* [54]. Unlike LpxH, LpxI does not utilize a catalytic metal center, although it did require divalent cations, specifically Mg^2+^, Mn^2+^, or Co^2+^, for optimal activity [54, 55]. Furthermore, ^18^O incorporation from H_2_^18^O indicated that LpxI catalyzes the nucleophilic attack of water on the *β*-phosphate of UDP-DAG [54].

**Figure 8:**
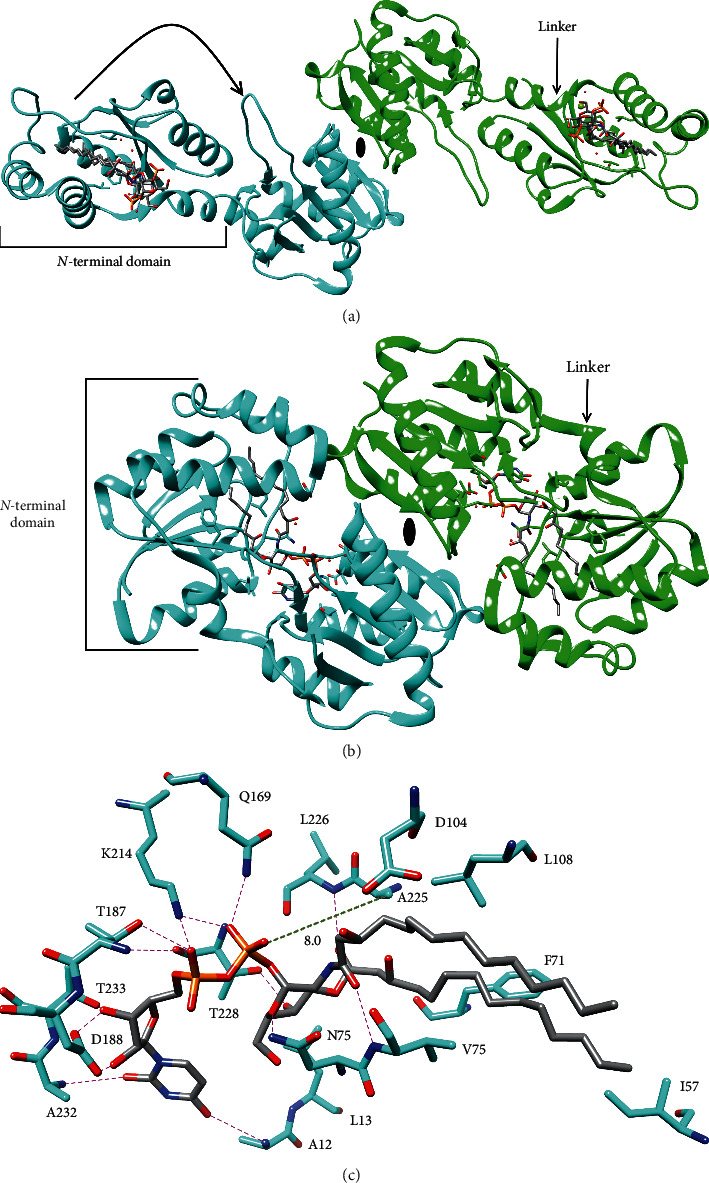
LpxI. (a) LpxI dimer bound to lipid X (PDB: 4GGM) [55]. (b) LpxI-D225 A dimer bound to UDP-DAG (PDB: 4J6E) [55]. (c) Residues involved in UDP-DAG binding to LpxI-D225 A from (b). Hydrogen-bond and salt-bridge interactions are shown, and the distance between A225 and the *β*-phosphate is highlighted. Distances in Ångstroms are as follows: 3.3 from 3′-acyl carbonyl to L226 N 2.5 from ribose 2′-hydroxyl to D188 O*δ*2, 2.3 from ribose 3′-hydroxyl to D188 O*δ*1 and 3.0 to T233 O*γ*, 3.3 from *α*-phosphate to T187 N and 2.8 to T187 O*γ* and 3.3 to K214 N*ζ*, 2.6 from *β*-phosphate to K214 N*ζ* and 2.9 to Q169 N*ε*, 2.5 from glucosamine 4′-hydroxyl to T228 O*γ*, 3.3 from uracil O2 to A232 N 2.9 from the glucosamine ring O to N74 N*δ*, 3.4 from uracil O4 to A12 N, and 3.1 from 2′-acyl carbonyl to V75 N.

Crystal structures of *Caulobacter crescentus* LpxI and LpxI-D225 A bound, respectively, to 2,3-bis(3R-hydroxymyristoyl)-glucosaminyl-1-phosphate (lipid X) and UDP-DAG revealed that LpxI has a novel two-domain fold wherein the *N*-terminal domain forms the binding pocket for the majority of the lipid substrate and the C-terminal domain forms the pyrophosphatase active site [55]. The *N*-terminal domain is composed of a parallel *β*-sheet surrounded by 4 *α*-helices, and the C-terminal domain is composed of a six-stranded *β*-sheet surrounded by 4 *α*-helices and a small two-stranded *β*-sheet [55]. The two domains are connected by a flexible linker that undergoes a secondary structure rearrangement to allow the domains to come together and bring the substrate into the active site (Figure 8) [55]. In the crystal structures, LpxI forms a dimer with two-fold symmetry (Figure 8), and LpxI also appeared to form a dimer in solution [55]. However, the functional relevance of dimerization is unclear [55].

In the structure of LpxI-D225 A in complex with UDP-DAG, uracil is bound by the backbone amides of residues 12 and 232, and ribose is hydrogen-bonded to D188 and T233 [55]. The pyrophosphate forms an ion pair with K214 and hydrogen-bonds with Q169 and T187 (Figure 8) [55].

Furthermore, the importance of T187 was supported by activity assays that showed T187 A had 175-fold less activity than wild-type LpxI [55]. Glucosamine is hydrogen-bonded to N74 and T288, and the carbonyls of the acyl chains are hydrogen-bonded to the backbone amides of residues 75 and 226, while the hydrocarbon chains bind in the complementary hydrophobic pocket of the *N*-terminal domain [55]. Although LpxI activity was stimulated by divalent cations, none are bound to the pyrophosphatase domain of either the crystal structure or are putative nucleophilic waters apparent [54, 55]. Therefore, the exact mechanism of catalysis remains unclear. However, positioning of A225 suggests that D225 may position the nucleophilic water for attack on the *β*-phosphate and/or act as the catalytic base, and in this case, the divalent cation may simply coordinate the unengaged side of the pyrophosphate to balance the negative charge to allow nucleophilic attack [55].

#### 2.1.7. LpxB

LpxB is the glycosyltransferase that forms the base glucosamine disaccharide of lipid A, catalyzing the formation of the glycosidic bond between the anomeric carbon of the UDP-DAG glucosamine and the 6-hydroxyl of lipid X to form lipid A disaccharide [56]. However, *lpxB* was first identified by a mutation that (in the presence of a mutation in *pgsA*) decreased synthesis of phosphatidylglycerol at 42°C [57]. *LpxB* mutants were also found to accumulate lipid X, a palmitoylated form of lipid X (lipid Y), and UDP-DAG, suggesting a role in lipid A synthesis, and furthermore, experiments with overexpressed and purified LpxB showed that LpxB forms the glucosamine disaccharide of lipid A [46, 56–61]. In addition, enzymatic assays showed that LpxB is diluted by increasing detergent concentration above the critical micelle concentration, which suggested that LpxB is a surface-active enzyme [61]. Despite being the founding member of the Raetz pathway, LpxB remained structurally uncharacterized until recently.

**Figure 9:**
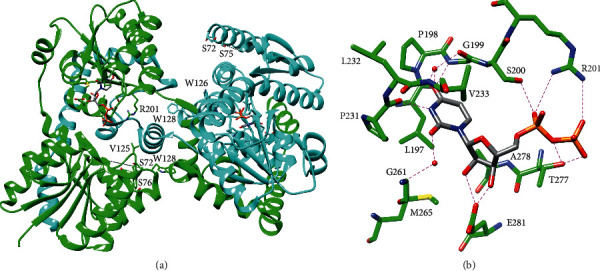
LpxB. (a) Ribbon structure of LpxB dimer bound to UDP (PDB: 5W8X) [62]. Three Ser side chains of six in the hydrophobic patch are shown; S66, S68, and S69 are part of preceding disordered loop and were not visible in the electron density [62]. V125, W126, and W128, which may line the hydrophobic groove that binds the substrates' acyl chains, are also shown [62]. (b) Residues involved in UDP binding from (a). Hydrogen-bond and salt-bridge interactions are shown. Distances of these interactions in Ångstroms are as follows: 2.5 from E281 O*ε*2 to ribose O2′ and 2.4 to ribose O3′, 2.9 from T277 O*γ* to *β*-phosphate O3 and 2.9 to pyrophosphate bridging O 3.8 from R201 N*η*1 to *α*-phosphate O2 and 3.8 from N*η*2 to *β*-phosphate O1, 2.9 from G261 N to water and 2.9 from this water to uracil O2, 2.5 from P231 O to uracil N3, 2.7 from G199 N to uracil O4, 3.0 and 2.8 from V233 N and G199 O (respectively) to water, and 3.2 from this second water to uracil O4.

The crystal structure of a solubilized form of *E. coli* LpxB, which was generated by the mutation of a surface-exposed hydrophobic patch, showed that LpxB forms a glycosyltransferase B (GT-B) dimer with a unique C-terminal swap wherein the last 87 residues of one subunit complete the fold of the opposite subunit (Figure 9) [62–64]. In total, the dimer is composed of four Rossmann-like domains with *β*-sheets surrounded by *α*-helices that are connected by *α*-helical linkers (Figure 9) [62]. The mutated hydrophobic patch appears to be part of a conserved membrane-association motif in GT-B enzymes that often consists of a hydrophobic loop followed by an amphipathic helix containing basic residues in the *N*-terminal domain [63]. This motif has been most well characterized in the GT-B PimA: the hydrophobic loop and basic residues were both found to be required for activity, and a Trp in the amphipathic helix was shown to associate with the membrane [65, 66]. Similarly, decreasing hydrophobicity of the surface-exposed patch in LpxB as hydrophobic residues was mutated to Ser correlated with decreasing activity [62].

The UDP-bound structure of LpxB was also solved, showing that the nucleotide-charged sugar donor substrate (UDP-DAG) binds to the C-terminal domain at the cleft between the domains as is typical for GT-B enzymes (Figure 9) [62–64]. The sugar acceptor substrate (lipid X) likely binds on the *N*-terminal side of the active site cleft near the predicted catalytic base (D98), and a hinge-like movement of the *N*-terminal domain, similar to conformational changes observed in other GT-B enzymes, may be required to bring the substrates into alignment for catalysis [61–64].

Uracil is recognized by hydrogen-bonding interactions with the backbone of residues 199 and 231 and via two water molecules to the backbone of residues 199, 233, and 261 (Figure 9) [62]. Ribose is recognized by E281, and the pyrophosphate is bound by hydrogen-bonds to S200 and T277 and a salt-bridge with R201 (Figure 9) [62]. R201 may serve to stabilize the negative charge of the UDP leaving group, and activity assays confirmed that this residue is critical for activity [61, 62]. Molecular docking of UDP-DAG correctly identified the UDP binding pocket and predicted that the acyl chains extend up a hydrophobic groove lined by V125, W126, and W128, and activity assays showed that W126 is indeed important for activity [61, 62]. Finally, the molecular docking model suggested that lipid X binds on top of UDP-DAG, which implies that LpxB follows an ordered sequential mechanism [62].

#### 2.1.8. LpxK

LpxK is the kinase that phosphorylates the 4′-hydroxyl of the distal glucosamine of lipid A disaccharide to form lipid IV_A_ [67, 68]. LpxK has been proposed to be the second regulatory point of the Raetz pathway: LpxK may be stimulated by unsaturated fatty acids, and its lipid A disaccharide substrate may stimulate the proteolysis of LpxC by FtsH, which requires the critical scaffold/chaperone protein LapB [33, 34]. This regulation allows Gram-negative bacteria to balance phospholipid and LPS synthesis as both pathways compete for *β*-hydroxymyristoyl-ACP [32]. High flux through the Raetz pathway depletes *β*-hydroxymyristoyl-ACP, limiting the production of unsaturated fatty acids, and decreased unsaturated fatty acid production leads to decreased LpxK activity, which causes a buildup of lipid A disaccharide [32, 33]. Buildup of this intermediate then leads to the proteolysis of LpxC, decreasing the consumption of *β*-hydroxymyristoyl-ACP by the Raetz pathway [32, 33]. In addition, LpxK is the final absolutely required step of the Raetz pathway as *E. coli* could transport lipid IV_A_ to the outer membrane and remain viable [7, 69].

LpxK was most active with ATP and an equimolar concentration of the divalent cation, showing little activity when divalent cations were removed with EDTA [67, 70]. For the lipid substrate, LpxK was specific for the glucosamine disaccharide head-group, showing no activity for lipid X, UDP-DAG, or substrates already containing Kdo core oligosaccharide sugars [68]. However, LpxK had little specificity for the number of acyl chains attached to the glucosamine disaccharide: LpxK could phosphorylate substrates with 2–6 acyl chains and substrates wherein the ester-linked chains were replaced with amide-linked chains [67, 68].

**Figure 10:**
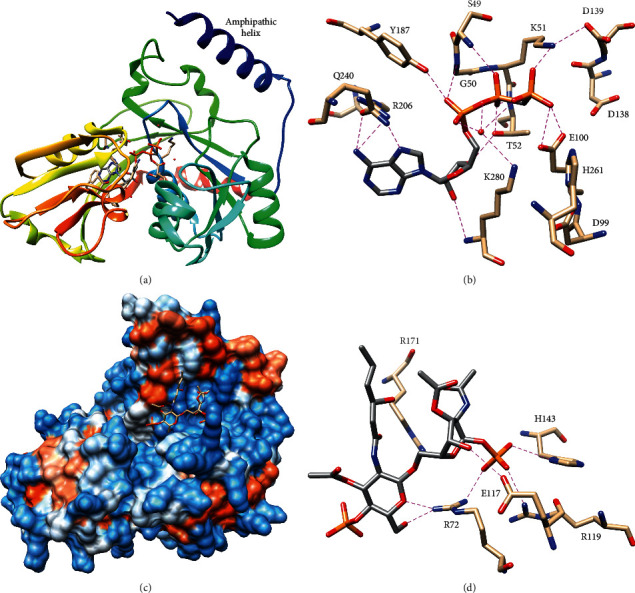
LpxK. (a) LpxK bound to AMP-PCP ATP analogue (PDB: 4ITL) [70]. Spectrum coloring begins with blue at the *N*-terminus. (b) Residues involved in AMP-PCP binding from (a). Distances for hydrogen-bond and salt-bridge interactions are shown with distances in Ångstroms as follows: 3.0 from adenine N6 to Q240 O*ε* and 3.5 to R206 N*η*1, 3.0 from adenine N7 to Q240 N*ε*, 2.8 from ribose 3′-hydroxyl to T52 O*γ*, 2.9 from *β*-phosphate to S49 N and 3.1 to K51 N and 2.7 to T52 N and 2.6 to T52 O*γ*, 3.0 from *α*-phosphate to G50 N and 2.6 to Y187 O*η*, 3.4 from ribose 2′-hydroxyl to K280 N 2.8 from D99 O*δ*1 to H261 N*δ*, 3.0 from K51 N*ζ* to D139 O*δ*1 and 2.7 to *γ*-phosphate, 2.9 and 2.8 from *γ*-phosphate to E100 O*ε*2/1, 3.2 from K280 N*ζ* to water, and 2.2, 3.0, and 3.0 from this water to the phosphates. (c) Hydrophobicity surface of LpxK bound to lipid IV_A_ (PDB: 4LKV) [72]. Hydrophobicity is shown in an orange-blue scale with blue representing the hydrophilic end. (d) Residues involved in binding lipid IV_A_ from (c). Distances in Ångstroms for hydrogen-bonds and salt-bridges are as follows: 2.4 from 6′-hydroxyl to R72 N*η*2 and 3.2 from the distal glucosamine ring O 3.4 from 1-phosphate to R119 N*η*1 and 3.2 to H143 N*δ* and 2.9 from R72 N*η*1, and 3.3 from 4-hydroxyl to E117 O*ε*.

Apo, substrate analogue-bound, and product-bound crystal structures of *Aquifex aeolicus* LpxK have been determined [70–72]. These structures reveal that LpxK is composed of two Rossmann-like domains with *β*-sheets surrounded by *α*-helices, and these domains are connected by two *β*-strands that are coextensive with the *β*-sheets of both domains (Figure 10) [71]. The larger *N*-terminal domain contains the P-loop/Walker A and Walker B motifs thus binding ATP, and the C-terminal domain binds the lipid substrate [70–72]. A hinge-like movement of the domains closes the distance between the binding pockets observed in the apo structure to bring the substrates into position for phosphoryl transfer [71]. In addition, LpxK has an *N*-terminal amphipathic helix and adjacent basic patch, which likely mediate membrane association; consistent with the presence of these structural elements, LpxK was optimally active at the critical micelle concentration of Triton X-100, and surface dilution of LpxK activity was observed at higher concentrations [70, 71].

Moreover, the amphipathic helix was required for activity [72]. LpxK was the first membrane active kinase of the P-loop-containing NTPase superfamily to be identified [71].

The AMP-PCP-bound structure of *A. aeolicus* LpxK shows that adenine is recognized by hydrogen-bonding to Q240 and R206, while ribose is bound by T52 and the backbone amide of K280 [70]. Furthermore, the *α*- and *β*-phosphates are engaged in extensive hydrogen-bonding interactions with T52, Y187, the backbone amides of residues 49–52, and an ordered water bound to K280 [70]. The *γ*-phosphate is positioned for transfer by a salt-bridge with K51 and hydrogen bonds to E100 and the ordered water (Figure 10) [70]. Homology with other P-loop-containing ATPases suggests that T52, E100, and D138 will coordinate Mg^2+^ in the complex with ATP-Mg [70, 71]. The role of E100 in Mg^2+^ coordination rather than simply in hydrogen-bonding is supported by E100 A/D/Q mutants, wherein E100Q showed the greatest decrease in *k*_cat_, and E100D showed the least as Asp can also coordinate Mg^2+^, and Ala may allow binding of a compensatory water [70]. In addition, H261 is positioned to accept a proton from the attacking 4′-hydroxyl of lipid A disaccharide, and D99, which is hydrogen-bonded to H261, likely serves to increase its pK_a_ [70]. Consistent with this role, D99 A/E/N mutants showed the same trend as the E100 mutants [70]. Kinetic experiments also confirmed the importance of K51, T52, and H261, showing 3000-fold decreases in *k*_cat_ for K51 A and T52 A and an 850-fold decrease for H261 A, and the Walker B motif residues D138 and D139 were observed to be critical for catalysis and ATP-Mg binding, decreasing *k*_cat_ 4700- and 8100-fold and increasing *K*_M_ 3.3- and 4-fold, respectively [70].

The structure of LpxK bound to its lipid IV_A_ product shows that LpxK binds the lipid IV_A_ glucosamines with the acyl chains extending into solvent and is largely disordered, which explains the minimal specificity for the extent of acylation [67, 68, 72]. The lipidic substrate binding site is on the same face as the *N*-terminal amphipathic helix, which suggests that LpxK may only partially extract lipid A disaccharide from the membrane during catalysis [72]. Even with glucosamine disaccharide, there are relatively few interactions [72]. The 1-phosphate is recognized by R72, R119, and H143; however, the proximal glucosamine is only hydrogen-bonded to E117, and the distal glucosamine is only hydrogen-bonded to R72 (Figure 10) [72]. Kinetic experiments confirmed the importance for lipid A disaccharide binding of R72 and H143, as Ala mutants increased *K*_M_ 4- and 7-fold, respectively, but R119 was largely dispensable, showing no change in *K*_M_ and only a 67-fold decrease in *k*_cat_ [72]. Finally, R171 A increased *K*_M_ 20-fold, but R171 was not directly bound to lipid IV_A_, which suggests that the lipid IV_A_-bound structure may not fully recapitulate productive lipid A disaccharide binding [72].

#### 2.1.9. WaaA (KdtA)

WaaA, also called KdtA, is the glycosyltransferase that, in *E. coli*, transfers two Kdo residues from CMP-Kdo: first to the 6′-hydroxyl of the distal glucosamine and then to the 4′-hydroxyl of the first Kdo [73, 74]. While *E. coli* WaaA is thus a bifunctional glycosyltransferase, WaaA variants from different species transfer 1–4 Kdo residues [1, 75, 76]. Activity assays also showed that, like LpxK, WaaA had little specificity for the extent of acylation, accepting substrates with 3–6 acyl chains, nor was 1-phosphate required [73, 74, 76]. However, 4′-phosphate was important as lipid A disaccharide was a poor substrate for *E. coli* WaaA although even lipid A disaccharide could be glycosylated to a lesser extent by *Aquifex aeolicus* WaaA [73, 74, 76]. Conversely, WaaA was highly specific for the donor substrate, showing activity and specific binding for CMP but no other nucleoside monophosphates [74, 76]. Interestingly, WaaA is another point of regulation in the Raetz pathway as WaaA was also degraded by the FtsH protease that targets LpxC [77].

**Figure 11:**
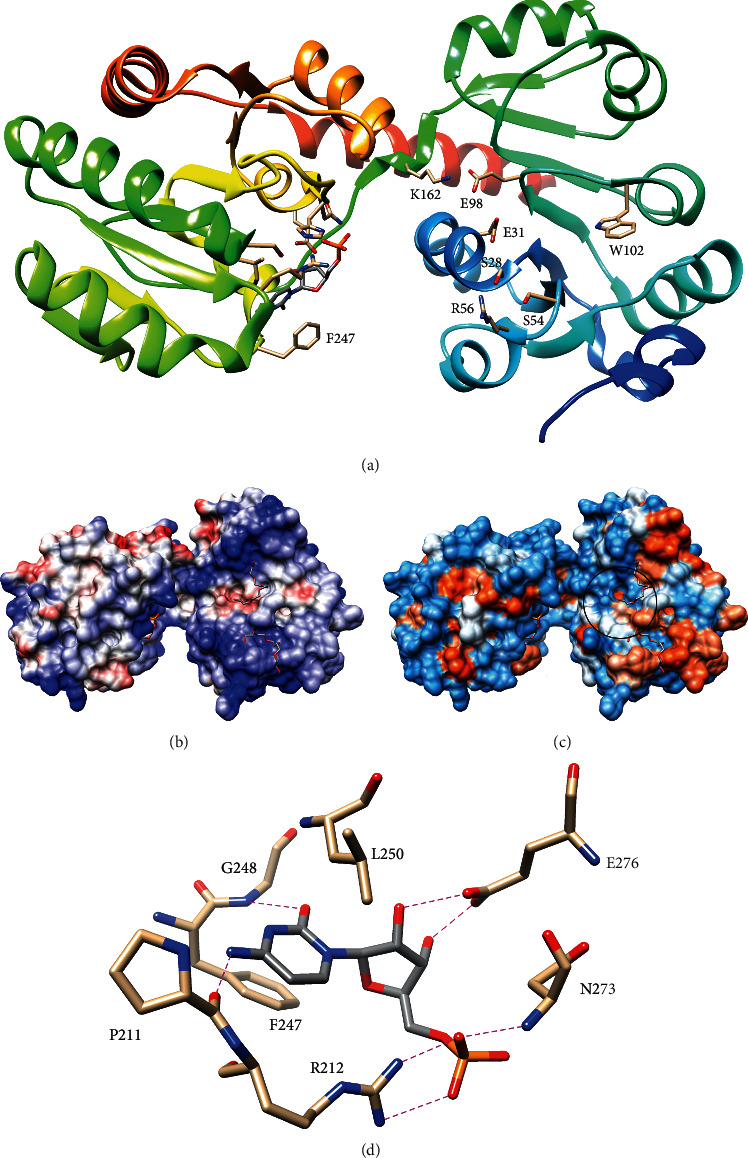
WaaA. (a) WaaA bound to the CMP product (PDB: 2XCU) [78]. Spectrum coloring ends with red at the C-terminus. (b) Coulombic surface of WaaA from (a). Charge is shown with a blue-red scale with blue at the positive end. Bound polyethylene glycol molecules are shown. (c) Hydrophobicity surface of WaaA from (a). Hydrophobicity is shown on an orange-blue scale with orange as the hydrophobic end. The putative lipid IV_A_-binding pocket is circled. (d) Residues involved in CMP binding from (a). Hydrogen-bond and salt-bridge interactions are shown with the following distances in Ångstroms: 3.0 from phosphate to N273 N and 2.6 and 3.2 to R212 N*η*1/2, 2.5 from E276 O*ε*1 to ribose 3′-hydroxyl and 2.8 to the 2′-hydroxyl, 2.9 from cytosine O2 to G248 N, and 3.1 from cytosine N4 to P211 O.

Crystal structures of the monofunctional WaaA from *A. aeolicus* revealed that WaaA has a typical glycosyltransferase B superfamily (GT-B) fold with two Rossmann-like domains (Figure 11) [78]. Moreover, the *N*-terminal domain of WaaA has a basic and hydrophobic surface as observed for other membrane surface-active GT-B enzymes (including LpxB as discussed above), which is thought to be required for productive membrane association [62, 63, 65, 66, 78, 79]. As expected for a GT-B enzyme, CMP was observed to bind to the C-terminal domain, and a putative lipid IV_A_ binding pocket was identified on the *N*-terminal domain [63, 78]. WaaA appears to have been crystallized in an open conformation with a wide cleft separating the domains; therefore, a hinge-like movement of the domains may be required to bring the substrates into position for nucleophilic attack [63, 65, 78–81].

The CMP-bound structure of *A. aeolicus* WaaA shows that cytosine binds in a hydrophobic pocket formed by F247 and L250 and is recognized by hydrogen-bonds with the backbone carbonyl of P211 and amide of G248 [78]. Ribose is hydrogen-bonded to E276, and the phosphate is engaged by R212 and N273 (Figure 11) [78]. Activity assays confirmed the importance of R212, which may stabilize the negative charge of the CMP leaving group during catalysis [78]. In addition, activity assays of Ala mutants confirmed the importance of residues G30, E31, E98, and K162 in the putative lipid IV_A_ binding pocket [78]. E31 appears well positioned to act as the catalytic base by accepting a proton from the 6′-hydroxyl of the distal glucosamine, and G30 could prevent steric clash from residue 30 that could interfere with proper alignment for nucleophilic attack [78]. If this role is correct for E31, then E98 and K162 may be important for positioning Kdo for transfer, possibly by contacting its 1′-carboxylate, and residues S28, S54, and R56 may bind the 1-phosphate of lipid IV_A_ [78]. Mutation of all 3 of these putative 1-phosphate binding residues to Ala significantly reduced activity [78]. Finally, increase in Trp-fluorescence upon lipid IV_A_ binding in the wild-type but not W102 A WaaA suggested that the acyl chains bind on the top of W102, shielding it from solvent quenching [78].

#### 2.1.10. LpxL and LpxP

In *E. coli*, LpxL is the acyltransferase that adds a lauroyl group to the *β*-hydroxyl of the 2′-*β*-hydroxymyristate on the distal glucosamine of Kdo_2_-lipid IV_A_ [82, 83]. Enzymatic assays showed that LpxL was a membrane surface active enzyme, and a predicted *N*-terminal transmembrane helix was required for activity *in vivo* [83]. In addition, enzymatic assays showed that LpxL was selective for the presence of the Kdo residues, as its activity for lipid IV_A_ was 6000-fold lower [83]. Furthermore, LpxL was selective for lauroyl-ACP: LpxL was 5% as active with lauroyl-coenzyme A (CoA), showed further reduced activity with decanoyl-CoA and myristoyl-CoA, and incorporated little or no acyl chains 16 carbons or longer from acyl-CoA [83].

Conservation of catalytic residues suggested that LpxL is related to the glycerol-3-phosphate acyltransferase (GPAT) family [83]. Consistent with this hypothesis, mutation to Ala of H132 and E137 of the predicted catalytic dyad in *E. coli* LpxL decreased activity 1000- and 3000-fold, respectively [83]. Moreover, Ala mutations of conserved R169 and D200 residues decreased activity 170- and 15-fold [83]. However, P238 A had little effect even though this Pro is important in the GPAT family, and this Pro was not conserved in the related enzyme LpxP [83].

*E. coli* also have a cold-shock inducible alternative to *lpxL* (*lpxP*) that replaces LpxL when cells are grown at 12°C [84]. LpxP transfers a palmitoleoyl chain from ACP instead of a lauroyl chain [84]. Like LpxL, LpxP was specific for the presence of the Kdo groups of Kdo_2_-lipid IV_A_ as LpxP failed to acylate lipid IV_A_ [84]. However, *E. coli* waaA mutants could produce penta- and hexa-acylated forms of lipid A containing laurate, myristate, and palmitoleate when grown on minimal media at 21°C, indicating that LpxL, LpxP, and LpxM have some activity for lipid IV_A_ [69]. In addition, LpxP was highly specific for (16 : 1) palmitoleoyl-ACP, showing little activity with shorter or saturated acyl chains or with palmitoleoyl-CoA [84]. While LpxL could substitute for LpxP at 12°C in a *δlpxP E. coli* strain, this strain showed increased sensitivity to rifampicin and vancomycin at 12°C, which suggests that incorporation of unsaturated acyl chains into the outer leaflet of the outer membrane by LpxP maintains the selective barrier at low temperature [85]. Neither LpxL nor LpxP have been structurally characterized, but the related enzyme LpxM has been [86].

#### 2.1.11. LpxM

In *E. coli*, LpxM is the acyltransferase that attaches a myristoyl group to the *β*-hydroxyl of the 3′-*β*-hydroxymyristate on the distal glucosamine of the LpxL product (Kdo_2_-lipid *V*_A_) to produce the end product of the Raetz pathway, hexa-acylated Kdo_2_-lipid A [87]. Enzymatic assays suggested that *E. coli* LpxM was specific for the penta-acylated substrate as LpxM acylated Kdo_2_-lipid IV_A_ and lipid IV_A_ much more slowly [87]. However, while LpxM showed poor activity for decanoyl-ACP, *β*-hydroxymyristoyl-ACP, palmitoyl-ACP, and palmitoleoyl-ACP, LpxM appeared to only have a slight preference for myristoyl-ACP over lauroyl-ACP [87].

The crystal structure of LpxM from *Acinetobacter baumannii* reveals that LpxM has a unique fold with a twisted, 7-stranded *β*-sheet surrounded by 10 *α*-helices that form a deep hydrophobic pocket and with an *N*-terminal transmembrane/membrane-association helix [86].

Unlike *E. coli* LpxM, *A. baumannii* LpxM transfers lauroyl chains to the *β*-hydroxyls of the acyl chains at the 2- and 3′-positions of the glucosamine disaccharide; therefore, *A. baumannii* LpxM is a bifunctional acyltransferase that transfers secondary lauroyl groups to both glucosamines to produce hepta-acylated lipid A [86]. Kinetic experiments showed substrate inhibition by lauroyl-ACP, suggesting that LpxM utilizes an ordered sequential mechanism in which the acyl acceptor must bind first [86]. Consistent with the bifunctionality of *A. baumannii* LpxM, substrate inhibition was best fit by a two-site binding model wherein only one lauroyl-ACP is inhibitory, and substrate inhibition was alleviated in the K282 E/K285 E double mutant of a putative ACP-binding site at the top of the lipid-binding pocket [86].

**Figure 12:**
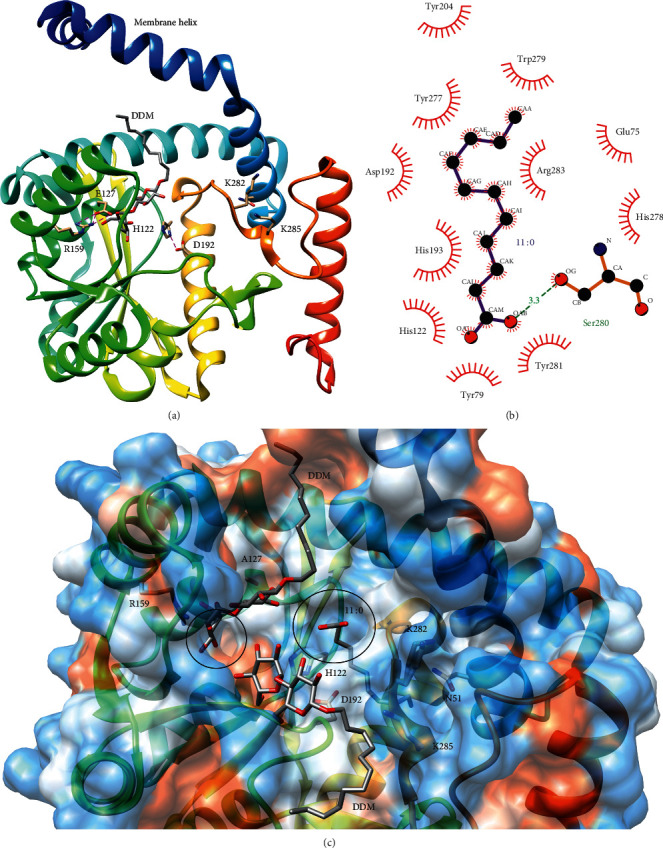
LpxM. (a) Ribbon diagram of LpxM (PDB: 5KN7) [86]. Spectrum coloring ends with red at the C-terminus. Interactions between putative catalytic residues are shown. Distances in Ångstroms are 2.6 from H122 N*δ* to D192 O*δ*1, 2.4 from E127 O*ε*1 to R159 N*η*2 and 3.4 to N*η*1, and 3.2 from E127 O*ε*2 to R159 N*η*2. (b) Ligplot+ diagram of fatty acid binding to LpxM (PDB: 5KNK) with distance shown in Ångstroms [45, 86]. Hydrophobicity surface of LpxM (PDB: 5KNK). Hydrophobicity is shown on an orange-blue scale with blue as the hydrophilic end. The entrances to putative hydrocarbon-ruler channels are circled.

Like LpxL, LpxM has a conserved His and Glu (H122 and E127 in *A. baumannii*) that, in distantly related acyltransferases, forms the catalytic dyad with the His acting as the catalytic base that accepts a proton from the nucleophilic hydroxyl [86, 88]. Kinetic experiments confirmed that E127 was critical for activity [86]. In addition, LpxM has a conserved Arg and Asp (R159 and D192); R159 was also confirmed to be critical for activity [86]. The crystal structure of *A. baumannii* LpxM reveals the location of these residues in the hydrophobic pocket, and the positions of these residues suggest that, at least in this bifunctional LpxM, the roles of the conserved HX_4_E/D motif have been separated to form two catalytic dyads that catalyze acyl transfer to the 2- and 3′-*β*-hydroxymyristoyl chains (Figure 12) [86]. In the glycerol-3-phosphate acyltransferase from *Cucurbita moschata*, the His and Asp of the HX_4_E/D catalytic dyad are hydrogen-bonded, suggesting that Asp increases the pK_a_ of His [89]. However, in *A. baumannii* LpxM, H122 and E127 are separated by 7.6 Å, and H122 is hydrogen-bonded to D192 [86]. Moreover, R159 forms an ion pair with E127 [86]. Therefore, H122 and D192 may serve as the catalytic dyad for the transfer of one lauroyl group, and E127 and R159 may act form the catalytic dyad for the transfer of the second lauroyl group. Finally, the structure of the *A. baumannii* LpxM E127 A mutant was resolved with an acyl chain (modeled as undecanoic acid) bound in one of two particularly deep, narrow channels in the active site (Figure 12) [86]. These channels may act as hydrocarbon rulers to control the length of the transferred acyl chains similar to those in LpxA and LpxD as discussed above [86]. Consistent with the hypothesis that H122/D192 and R159/E127 form two catalytic dyads for separate acyl transfer reactions, one of these putative hydrocarbon-ruler channels is near H122 and D192, and the other channel is near R159 and E127 (Figure 12).

#### 2.1.12. LpxJ and LpxN

Some bacteria encoding LpxL but not LpxM instead encode a distant homologue of LpxL-, LpxM-, and LpxP-designated LpxJ [90]. LpxJ enzymes from three *ε*-proteobacteria species: *Helicobacter pylori*, *Campylobacter jejuni*, and *Wolinella succinogenes*, which were characterized, showing a range of substrate specificities [90]. Ultraviolet photon dissociation mass spectrometry combined with activity assays confirmed that *H. pylori* LpxJ acylated the *β*-hydroxyl of the 3′-acyl chain, like *E. coli* LpxM, and not the acyl chains of the proximal glucosamine [90]. However, none of these enzymes required the presence of the Kdo groups, and *H. pylori* LpxJ was not specific for the presence of the 2′-secondary acyl chain added by LpxL, while the other LpxJ variants were only active on Kdo_2_-lipid IV_A_ or lipid IV_A_, not the LpxL product [90]. *H. pylori* and *W. succinogenes* LpxJ were most active with lauroyl-ACP, but *H. pylori* LpxJ also utilized myristoyl-ACP [90]. On the contrary, *C. jejuni* LpxJ was most active with palmitoyl- or stearoyl-ACP [90]. In addition, *Vibrio cholerae* utilizes another alternative to LpxM, designated LpxN, that transfers a *β*-hydroxylauroyl chain to the *β*-hydroxyl of the 3′-acyl chain [91]. Thus, the different activities and substrate specificities of the LpxL/M homologues encoded by different bacterial species result in diversity in the length, extent, and position of acylation in the final lipid A product of the Raetz pathway in different species [1].

### 2.2. LPS Synthesis and Transport

#### 2.2.1. Core Oligosaccharide Synthesis

**Figure 13:**
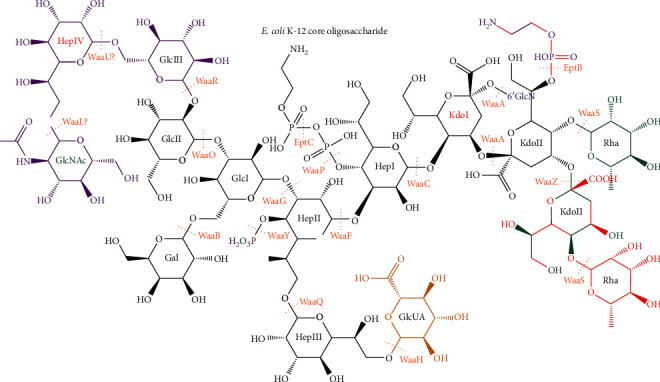
Core oligosaccharide synthesis. Coloring of core oligosaccharide is the same as in Figure 1. Transferases responsible for the formation of bonds linking residues in the K-12 core oligosaccharide are marked in orange [1, 3, 4, 95–100].

Following the completion of the Raetz pathway, the core oligosaccharide is completed at the cytoplasmic side of the inner membrane [1, 92]. The core oligosaccharide can be divided into inner and outer cores, and the inner core tends to be conserved within a species or genus, while species typically have a small number of different outer cores synthesized in different strains [1, 92]. Because of the variability of core oligosaccharides, a discussion of the biochemical and structural details of biosynthetic enzymes that synthesize the many different core oligosaccharides is beyond the scope of this review. For an overview of the structure and biosynthesis of different core oligosaccharides, the reader is referred to the following reviews [1, 93, 94]. However, the biosynthetic pathway of the core oligosaccharide from *E. coli* K-12 is briefly covered as an example. As described above, the first two Kdo residues are added during the Raetz pathway, and these sugar residues are the most conserved moiety of the inner core with all known core oligosaccharides containing Kdo [1, 73, 74]. In addition, the inner core typically contains L-glycero-D-mannoheptose (Hep) or, less frequently, the D-glycero-D-mannoheptose isomer [1]. In *E. coli*, the first and second Hep residues (Figure 13) are added sequentially to the first Kdo by WaaC and WaaF, respectively, and a branching Hep is added to the second Hep by WaaQ [1, 95]. The first and second Hep residues are phosphorylated by WaaP and WaaY, respectively, and the activity of these kinases is dependent on the addition of the first outer core residue by WaaG though WaaP retained some activity in *δwaaG* strains [1, 96, 101].

These phosphorylated inner core residues are thought to be important for crosslinking of LPS molecules by divalent cations, and loss of phosphorylation increases sensitivity of *E. coli* to detergent and some antibiotics and strongly induces RpoE-regulated envelope stress response via increased expression of this *σ* factor [1, 96, 101]. Moreover, the second Hep is the attachment site of this first outer core residue, which is glucose in *E. coli* [1, 97]. In *E. coli* K-12, the first glucose is the site of attachment of a branching galactose by WaaB and a second glucose residue by WaaO [97]. This WaaO product is a branch point in core synthesis in *E. coli* K-12: either WaaR adds a third glucose to the second glucose, or WaaZ adds a third Kdo to the second Kdo [3]. Competition between these pathways appears to be controlled by the relative levels of WaaR and WaaZ, which are dependent on growth conditions with phosphate limitation favoring WaaZ and optimal growth conditions favoring WaaR [3]. Following addition of the third Kdo, WaaS can attach L-rhamnose (Rha) to the second Kdo or, if the second Kdo has already been modified with phosphoethanolamine by EptB, to the third Kdo [3]. In the WaaR pathway, a fourth heptose is attached to the third glucose to complete the outer core (Figure 13) [1]. Sequence similarity with other heptosyltransferases suggests that this reaction is catalyzed by WaaU [99].

#### 2.2.2. Transport of Core-lipid across the Inner Membrane by MsbA

**Figure 14:**
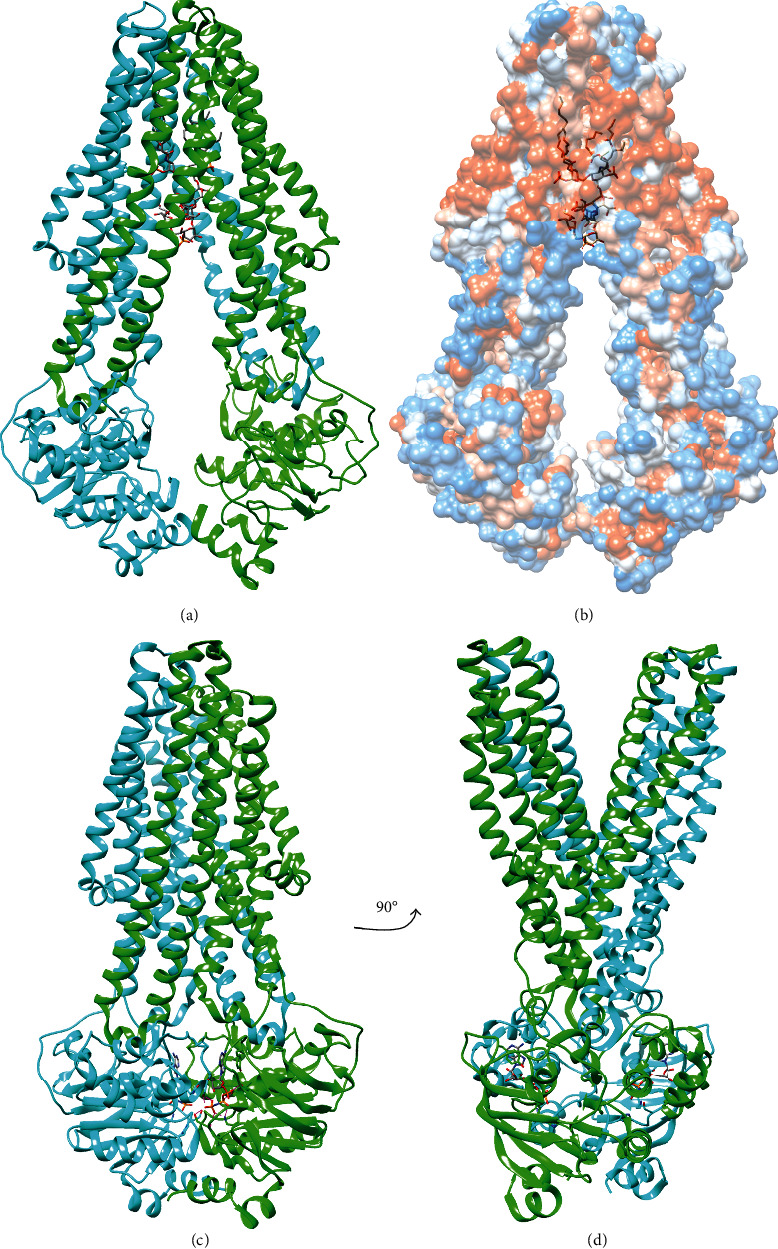
MsbA. (a) Cytoplasmic-open conformation of MsbA bound to LPS (PDB: 5TV4) [104]. (b) Hydrophobicity surface of MsbA in (a). Orange is the hydrophobic end of the scale, and blue is the hydrophilic end. The mostly orange region roughly indicates the coverage of the nanodisc membrane in the electron density [104]. (c) Periplasmic-open conformation of MsbA bound to AMP-PNP ATP analogue (PDB: 3B60) [103].

Following the completion of the core oligosaccharide, the core-lipid A molecule is flipped across the inner membrane by the ATP-binding cassette (ABC) transporter MsbA [7, 92, 102]. Decreased activity of MsbA for earlier intermediates of LPS synthesis lacking core sugars or secondary acyl chains likely helps to prevent premature transport [7]. Structures of MsbA obtained by cryogenic electron microscopy (cryo-EM) and X-ray crystallography have provided insights into the mechanism of transport and selectivity [103–105]. These structures show that MsbA forms a functional dimer wherein the transmembrane domains are comprised of 4 transmembrane helices from one subunit and 2 transmembrane helices from the opposite subunit (Figure 14) [103, 104]. A cytoplasmic P-loop containing the ATPase domain is appended to TM6, and cytoplasmic helices between TM2 and TM3 and between TM4 and TM5 contact the ATPase domains [103, 104]. The helix between TM2 and TM3 contacts its own ATPase domain at the ATP-binding site, and the helix between TM4 and TM5 contacts the ATPase domain of the opposite subunit (Figure 14) [103, 104].

The ATPase domains also act as a functional dimer: the 3.7 Å crystal structure of *Salmonella* MsbA bound to a nonhydrolyzable ATP analogue (AMP-PNP) shows that ATP binds between the ATPase domains (Figure 14(c)) [103]. One half of the ATPase active site formed by the interstrand loops of two *β*-sheets and an *α*-helix sandwiched between them from one ATPase domain, and the other half is contributed by the *α*-helical side of the other ATPase domain [103]. In this structure, the transmembrane domain assumes a periplasmic open conformation (Figure 14(c)) [103].

A 4.2 Å cryo-EM structure of *E. coli* MsbA in the cytoplasmic open conformation shows core-lipid A bound inside the transmembrane domains with partially resolved acyl chains and core oligosaccharide resolved out to the three inner core Hep residues (Figure 14) [104]. The core-lipid A binding pocket is separated into hydrophobic lipid A-binding and hydrophilic oligosaccharide-binding regions by a ring of primarily basic residues that recognize the phosphorylated glucosamine disaccharide of lipid A [104]. Additionally, a recent 2.9 Å crystal structure of MsbA bound to core-lipid A and a small molecule inhibitor fully resolved the acyl chains, and the tight packing of the chains into the hydrophobic pocket suggests that MsbA selectivity for completed core-lipid A may be partially contributed by packing of acyl chains of the correct length and number [105].

The acyl chains are directed toward the periplasm suggesting that flipping occurs after a conformational change to open the periplasmic side of MsbA; however, examination of the hydrophobicity of MsbA (Figure 14(b)) suggests that the requisite translation has already occurred with the acyl chains already extending to the periplasmic leaflet of the inner membrane [104]. A second 4.8 Å cryo-EM structure, determined in the presence of ADP and vanadate, shows MsbA in an occluded conformation with parallel transmembrane helices [104]. Comparison of the structures shows that transition of the transmembrane domains from cytoplasmic-open to periplasmic-open involves a shuffling of the TM helices that may help flip core-lipid A [104].

Ergo, the structures of MsbA suggest a mechanism, wherein core-lipid A binds the cytoplasmic open conformation, and ATP binding brings the ATPase domains together, which is coupled to a conformational change in the transmembrane domains from cytoplasmic open to periplasmic open [103, 104]. Core-lipid A flips and diffuses into the periplasmic leaflet [103, 104]. Next, ATP hydrolysis is coupled to a periplasmic open to occluded conformational change, which prevents the transporter from acting in reverse [104]. Finally, release of phosphate allows MsbA to return to the starting cytoplasmic open conformation [103, 104].

Recently, a set of small molecules that selectively inhibit MsbA core-lipid A transport and ATP hydrolysis were identified, and the binding of two were characterized by X-ray crystallography [105]. These inhibitors were found to bind simultaneously with core-lipid A in an adjacent membrane-exposed cleft formed by TM4-6 of each subunit in the cytoplasmic open conformation [105]. Because this pocket will be deformed by the transition to the periplasmic open conformation, these inhibitors may lock the MsbA transmembrane domains into the cytoplasmic open conformation [105]. Moreover, inhibitor binding appeared to strain TM4 leading to a movement of the ATPase domains via the coupling cytoplasmic helix between TM4 and TM5 [105]. The ultimate result of this inhibitor-induced conformational change was the partial detachment of one ATPase domain from its corresponding coupling cytoplasmic helices and the loss of the symmetrical arrangement of the ATPase domains required for ATP hydrolysis, which suggests that these inhibitors act by locking MsbA in a catalytically inactive state [105].

#### 2.2.3. O-Antigen Polysaccharide Synthesis and Transport

The O-antigen is a repeating polysaccharide that varies between bacterial strains [1]. Different O-antigens are synthesized by one of three divergent pathways: Wzy dependent, ABC transporter dependent, and synthase dependent [1, 92]. However, O-antigen synthesis has a few conserved features: O-antigen is built on undecaprenol phosphate (und-P), and sugar residues are transferred to this lipid from nucleotide-charged sugar donors at the cytoplasmic face of the inner membrane [1, 92]. Furthermore, a sugar-1-phosphate [1-phospho-*N*-acetyl-glucosamine (P-GlcNAc) in *E. coli*] is transferred to und-P to form a phosphoanhydride product (GlcNAc-PP-und in *E. coli*) [1]. Finally, the completed O-antigen is ligated to core-lipid A at the periplasmic face of the inner membrane to form completed LPS by WaaL, which is specific for core oligosaccharides but not O-antigens [1]. The lack of polysaccharide specificity of WaaL also allows it to attach other capsular polysaccharides (K-antigens and colonic acid in *E. coli*) to core-lipid A [100, 106]. Moreover, while *E. coli* K-12 does not synthesize O-antigen, conservation of the machinery shared with other polysaccharide synthesis pathways still enables this strain to synthesize GlcNAc-PP-und and transport it across the inner membrane [99, 106, 107]. Therefore, WaaL may also transfer *N*-acetyl-glucosamine to the terminal Hep of the K-12 outer core (Figure 13) [99, 100]. Studies of the topology of WaaL indicated that the ligase contained 12 transmembrane segments and 2 major periplasmic segments, and enzymatic assays showed that a conserved Arg in a short periplasmic segment and a conserved His in the largest periplasmic segment were essential for activity [108, 109]. Examination of the reactants and products of the WaaL reaction suggest that WaaL catalyzes an S_N_2 substitution reaction, wherein the terminal residue of the core oligosaccharide (7′-hydroxyl of the fourth Hep in *E. coli* K-12) attacks the anomeric carbon of the basal residue of the O-antigen with und-PP acting as the leaving group [109]. The positions of these residues and the metal-independent activity of WaaL make it tempting to speculate that the conserved Arg and His could bind the pyrophosphate of O-antigen-PP-und and act as the catalytic base for the attacking hydroxyl, respectively [108, 109].

The Wzy dependent pathway is unique from the other pathways, and in that single repeat units are synthesized and transported across the inner membrane [1]. Once the repeat unit has been synthesized by its specific glycosyltransferases, the repeat unit-PP-und is flipped across the inner membrane by a Wzx flippase [110]. Little is known about the mechanism of Wzx flippases; even whether they require energy is a matter of contention though *in vitro* transport of a soluble analogue of GlcNAc-PP-und was not affected by the addition of ATP, NADH, or ionophores [107, 110]. However, Wzx flippases are thought to have 12 transmembrane segments, and they tend to be specific for a particular O-antigen repeat unit with the first sugar attached to und-PP playing the most important role in substrate specificity [108, 110]. Next, the O-antigen repeat units are polymerized at the periplasmic face of the inner membrane on a single und-PP by Wzy, which catalyzes the transfer of the growing polysaccharide to the free end of the new repeat unit-PP-und [1]. Topology studies of Wzy from *Pseudomonas aeruginosa* PAO1 suggested that the polymerase contained 14 transmembrane segments and 4 major periplasmic segments [108]. Two of these periplasmic segments were found to contain RX_10_ G motifs with highly conserved Arg residues that were essential for activity [111, 112].

**Figure 15:**
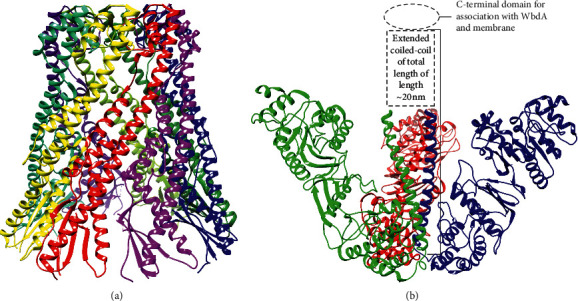
Proteins that control O-antigen modal length. (a) Octamer of WzzB periplasmic domains (PDB: 4E29) [113]. (b) WbdD trimer (PDB: 4UW0) with the location of the unresolved extended coiled-coil domain and uncharacterized C-terminal domain marked by a rectangle and oval, respectively [114, 115].

The presence of two similar motifs in Wzy corresponding with two similar substrates and the conservation of Arg suggests that the Arg residues could be involved in binding a common motif in Wzy substrates such as the pyrophosphate of oligo-PP-und [108, 111, 112]. In addition to a specific sugar composition, O-antigens have a strain specific modal length, and this is controlled by Wzz [1, 113, 116]. While crystal and cryo-EM structures of Wzz length regulators from multiple species have been determined, the mechanism of length regulation has remained obscure [113, 116, 117]. Wzz is primarily a periplasmic protein with *N*- and C-terminal transmembrane helices, and the periplasmic region forms bell-shaped oligomers with 5–12 subunits though open trimers have also been observed [113, 116, 117]. The periplasmic region has an *α*/*β*-domain that forms the base of Wzz adjacent to the membrane and an extended *α*-helical domain that forms a twisted helix bundle in oligomers (Figure 15) [113, 116, 117]. *In vivo* assays of Wzz mutants and chimeras indicated that surface-exposed residues at the top of the *α*-helical domain controlled O-antigen length, but the role of oligomerization in length regulation (if any) has not been established [113, 116].

In the ABC transporter dependent pathway, the O-antigen is synthesized continuously on a single und-P carrier at the cytosolic face of the inner membrane [1, 118]. Consequently, the initial 1-phospho-sugar and second adaptor residue are not repeated, forming the adaptor region before the repeat region of the O-antigen [1, 118]. Moreover, ABC transporter-dependent O-antigens can contain terminal modifications to the free end of the O-antigen that act as a form of quality control to ensure only completed O-antigen is transported across the inner membrane [1, 118, 119]. In terminally modified O-antigens synthesized by the ABC transporter dependent pathway, the strain specific modal length of the O-antigen is controlled by the enzyme that makes the terminal modifications, which halt the activity of the glycosyltransferases that add the repeat units [114, 118]. Crystal structures and small angle x-ray scattering (SAXS) data of terminating enzyme of *E. coli* O9a (WbdD) revealed that the catalytic domain of WbdD is separated from a structurally uncharacterized C-terminal domain by an extended *α*-helical domain that forms a ∼200 Å coiled-coil in WbdD trimers (Figure 15) [114]. The C-terminal domain mediated association with the membrane and the glycosyltransferase that synthesizes the O9a repeat units (WbdA) [115]. Deletions that decreased the length of the coiled-coil domain decreased the modal length of the O-antigen *in vivo*, and the opposite effect was observed for insertions designed to increase the length of the coiled-coil [114]. Therefore, these data support a model wherein O-antigen is lengthened by WbdA until long enough to reach from the membrane to the catalytic domain of WbdD at which point termination depends on competition between WbdA and WbdD for the free end of O-antigen [114]. However, some ABC transporter-dependent O-antigens lack terminal modifications, and in this case, the modal length is controlled by the stoichiometry of the glycosyltransferases that synthesize the repeat units and of the ABC transporter [118]. Finally, the completed O-antigen is transported across the membrane by the ABC transporter [1, 118].

**Figure 16:**
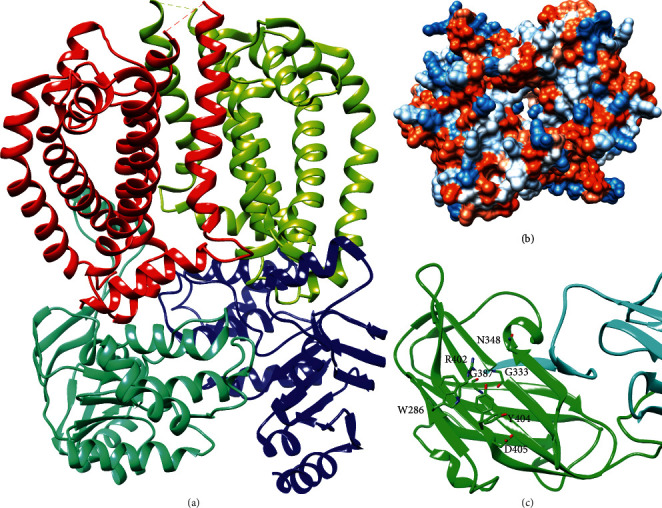
O-antigen ABC transporter. (a) Wzm-Wzt heterotetramer ABC transporter from *Aquifex aeolicus* (PDB: 6AN7) [120]. The Wzm subunits are shown in red and yellow, and the ATPase domain of the Wzt subunits are shown in cyan and blue. (b) Hydrophobicity surface of Wzm-Wzt heterotetramer from (a) showing periplasmic end of the continuous channel. Hydrophobicity is shown on an orange-blue scale with orange as the hydrophobic end. The minimum radius of the channel is 3.5 Å [120]. (c) C-terminal carbohydrate-binding domain of Wzt from *E. coli* O9a (PDB: 2R5O) [119]. The domain was crystallized as a C-terminally swapped dimer, but this C-terminal swap may not occur in the full Wzm-Wzt complex. Residues found to be involved in specific recognition of terminally modified O9a O-antigen are shown [119].

The structure of the heterotetramer complex of the Wzm permease and the *N*-terminal ATPase domain of Wzt from *Aquifex aeolicus* and the structure of the C-terminal carbohydrate-binding domain of Wzt from *E. coli* O9a have been solved by X-ray crystallography [119, 120]. The Wzm-Wzt ABC transporter complex was solved in an open conformation and forms a continuous channel with a minimum radius of 3.5 Å, suggesting that the polysaccharide can occupy the channel through multiple rounds of ATP hydrolysis (Figure 16) [120]. The Wzm channel is lined with several aromatic residues that may form stacking interactions with the sugar rings [120]. The Wzt C-terminal domain forms an immunoglobulin-like *β*-sandwich with a groove containing several residues that were shown to be involved in recognition of the terminally modified O9a polysaccharide *in vivo* and *in vitro* (Figure 16) [119].

As in the ABC transporter-dependent pathway, synthase-dependent O-antigen is synthesized on a single und-P carrier and thus contains an unrepeated adaptor region [1]. The synthase (WbbF) is thought to simultaneously add repeat units and transport the growing O-antigen across the membrane [1]. However, only O:54 O-antigen of *Salmonella enterica* is known to be synthesized by this pathway, and it has thus not been studied extensively [1].

#### 2.2.4. Transport of LPS to the Outer Membrane

Completed LPS is transported from the periplasmic leaflet of the inner membrane to the outer leaflet of the outer membrane by the Lpt complex, which is comprised of proteins LptA-G and spans from the cytoplasmic face of the inner membrane to the extracellular face of the outer membrane [121–124]. This complex, which is formed by *β*-jellyroll domains in LptF, LptG, LptC, LptA, and LptD, forms a continuous hydrophobic slide for LPS across the periplasm, and transport is driven by the ATPase activity of LptB [123–132]. Extensive structural and functional data have been obtained to support this transenvelope protein bridge mechanism.

**Figure 17:**
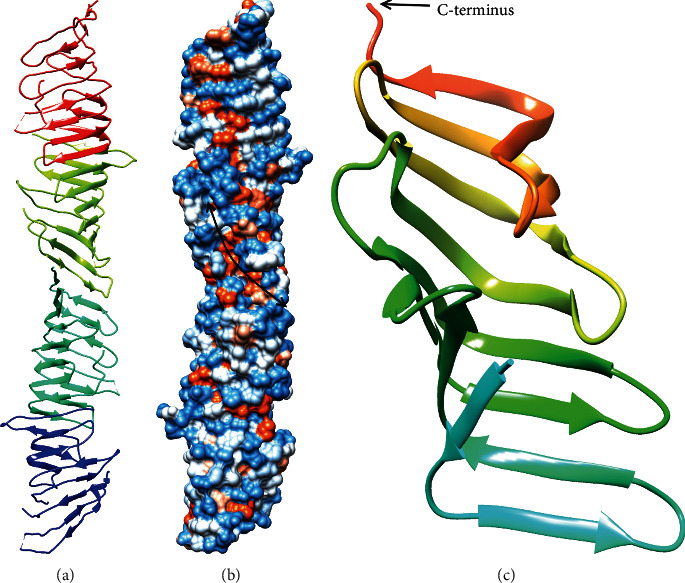
LptA and LptC. (a) LptA tetramer with subunits interacting in a head-to-tail fashion (PDB: 2R1A) [129]. The tetramer is shown with the *N*-terminal end at the bottom. (b) Hydrophobicity surface of LptA tetramer in (a). Hydrophobicity is shown on an orange-blue scale with blue at the hydrophilic end. The arrow indicates the continuous, helical, and hydrophobic groove [129]. (c) Ribbon structure of LptC periplasmic domain (PDB: 3MY2) [130].

Some of the earliest functional evidence for the transport of LPS via a transenvelope bridge was obtained by the study of lipid transport from inner membrane spheroplasts [133]. These spheroplasts were found to contain pieces of the outer membrane that could be separated by centrifugation [133]. Newly synthesized (^14^C-labeled) LPS was transported to the outer membrane in an MsbA-dependent, periplasmic extract-independent manner, and this contrasted with the transport of lipoproteins, which could be released from spheroplasts by the addition of the soluble periplasmic carrier protein LolA, and with the transport of newly synthesized phospholipids, which could not be transported to the outer membrane outside of intact cells [133]. Further studies utilized lipid A modifying enzymes to demonstrate that LPS accumulated at the periplasmic face of the inner membrane when the soluble periplasmic protein LptA was inactivated thus identifying a putative LPS carrier for this process [121]. The idea of a protein bridge was further supported by crystal structures of LptA [129]. The crystal structures revealed that LptA is primarily comprised of two 8-stranded *β*-sheets that fold into a *β*-jellyroll with a hydrophobic cleft at one edge (Figure 17). Moreover, in the presence of LPS (though LPS is not visible in the electron density), LptA crystallized as a chain of head-to-tail interacting subunits that formed two continuous *β*-sheets and a continuous hydrophobic groove suggestive of a hydrophobic slide for LPS [129].

Next, the crystal structure of the LptC periplasmic domain showed a very similar structure to that of LptA (Figure 17) supporting the model of a continuous *β*-jellyroll slide for LPS [130]. Moreover, LptA could abstract LPS from LptC but not vice versa thus supporting affinity-based directional transport between proteins in the Lpt system, though these results are also consistent with a soluble carrier mechanism of LptA [130]. However, the stable protein bridge model was supported by the ability of the cytosolic protein LptB and inner membrane proteins LptC and LptF to pull down LptA and the outer membrane proteins LptD and LptE and by the ability of LptA to cause the association of liposomes containing the LptBCFG complex with liposomes containing the LptDE complex [122, 124].

Interactions between the Lpt proteins were further characterized by site-specific crosslinking with the nonnatural amino acid *p*-benzoyl-L-phenylalanine (Bph) [127]. Crosslinking suggested that the C-terminal end of the LptC *β*-jellyroll interacted with the *N*-terminal end of the LptA *β*-jellyroll, and the C-terminal end of the LptA *β*-jellyroll interacted with the *N*-terminal end of the LptD *β*-jellyroll [127]. In addition, C-terminal mutants of the LptC *β*-jellyroll were found to be nonfunctional *in vivo* and unable to pull down LptA, LptD, or LptE [125, 126]. On the contrary, the *N*-terminal transmembrane domain of LptC (a predicted transmembrane helix) seemed to be of limited functional importance as it could be functionally replaced by unrelated transmembrane helices [125].

Crosslinking with Bph was also utilized to provide evidence for binding and directional transfer of LPS [123, 124]. LPS was crosslinked to LptA and LptC when Bph was incorporated in the hydrophobic cleft of their *β*-jellyrolls but not when Bph was incorporated on the outer surface of their *β*-jellyrolls thus supporting transport along a continuous hydrophobic slide [123, 124]. In addition, ATP-dependent transfer of LPS from LptBFG to LptC and LptA was observed *in vivo* and in liposomes, and transfer to LptA was enhanced by the inclusion of LptC [123, 124]. Furthermore, LPS was transferred from liposomes containing LptBCFG to liposomes containing LptDE in an ATP- and LptA-dependent manner [124]. Moreover, increase in ATP concentration resulted in LPS crosslinking to LptD at earlier time points and decreased crosslinking to LptD at later time points, which strongly indicated that transfer through the Lpt system was an active process that could be accelerated by an increased rate of ATP hydrolysis by LptB [124]. Therefore, these data support a model, wherein each new LPS molecule pushes the previous LPS molecules along the hydrophobic slide of the Lpt complex until they reach the outer leaflet of the outer membrane [124].

Crystal structures of the LptB_2_FG ABC transporter and of the LptB ATPase dimer bound to ATP have been solved and provide some insights into how ATP hydrolysis drives LPS through the Lpt system [128, 132, 134].

**Figure 18:**
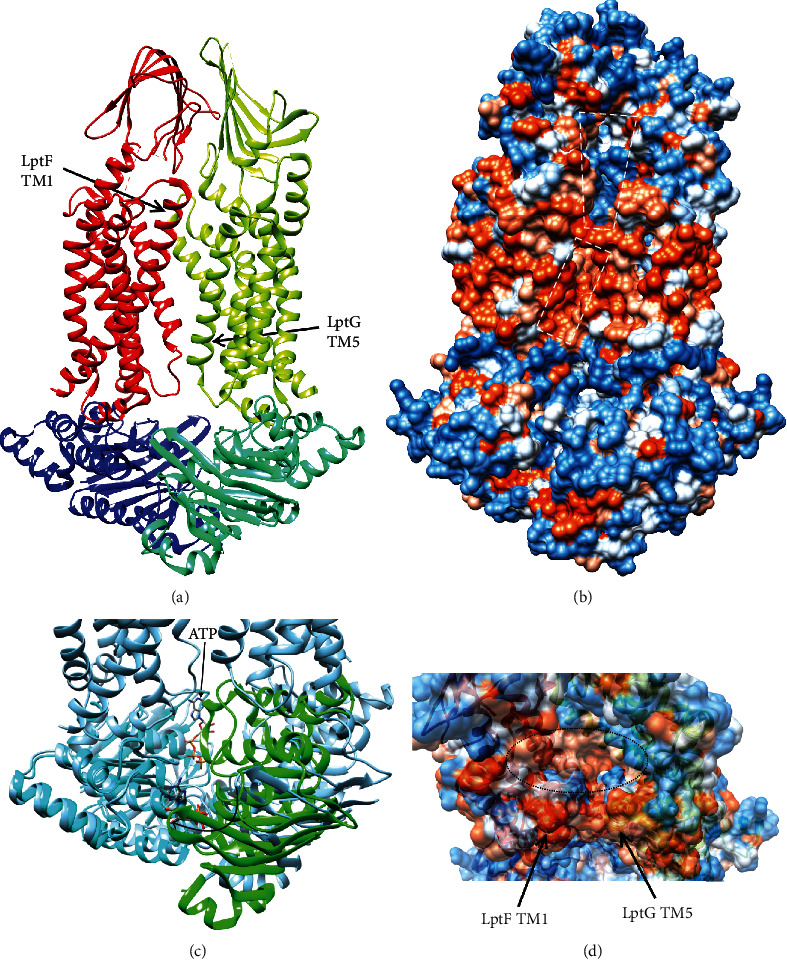
LptB2FG ABC transporter. (a) Ribbon structure LptB_2_FG ABC transporter (PDB: 5X5Y) [128]. LptF is shown in red, while LptG is shown in yellow, and LptB subunits are shown in cyan and blue. (b) Hydrophobicity surface of (a). Hydrophobicity is shown on an orange-blue scale with orange as the most hydrophobic. Quadrilaterals mark spaces between LptF TM1 and LptG TM5. (c) Comparison of ATP-bound LptB dimer (PDB: 4P33) [134] with LptB subunits in nucleotide-free LptB_2_FG complex from (a). Subunits from ATP-bound LptB-E163Q dimer are colored cyan and green, and the LptB_2_FG complex is colored light blue. Cyan LptB-E163Q was overlaid with LptB chain B of complex [24]. Curved arrow shows movement of other LptB subunits from nucleotide-free conformation in LptB_2_FG complex to ATP-bound conformation in the LptB-E163Q dimer. (d) Rotated view of (b) showing opening to V-shaped pocket on top/periplasmic side of LptB_2_FG complex. Ellipse marks the opening.

The structure of the LptB_2_FG complex was solved in the nucleotide-free state and shows that the transmembrane helices of LptF and LptG form a V-shaped pocket at the periplasmic face of the inner membrane (Figure 18) [128, 132]. There are minimal contacts at the interface between TM1 of LptF and TM5 of LptG suggesting that these helices may separate to allow LPS to enter by lateral diffusion (Figure 18) [128, 132]. The V-shaped pocket is lined by highly conserved hydrophobic residues that were found to be important for function *in vivo* [128]. In addition, LptF and LptG have periplasmic *β*-jellyroll domains similar to those of LptA, LptC, and LptD [128–132]. Mutation of the C-terminal strands of the LptF or LptG *β*-jellyroll domains caused growth defects in *E. coli*, which suggested that these domains may interact with the *N*-terminal end of the LptC *β*-jellyroll to form the first unit in the hydrophobic slide [132].

The LptB-E163Q dimer was crystallized with ATP bound showing that ATP binds between the ATPase subunits (Figure 18) [134]. E163 is the predicted catalytic base, and mutation to Gln eliminated ATPase activity [134]. Comparison of the ATP-bound LptB dimer and the nucleotide-free LptB dimer of the LptB_2_FG ABC transporter suggests that ATP binding to the transporter will cause the LptB subunits to rotate ∼15° [128, 134]. Interactions of LptB with TM1 and the short *α*-helix between TM2 and TM3 of LptF and LptG likely serve to transmit this motion into the further opening of the space between LptF TM1 and LptG TM5 to allow LPS binding [128]. Other conformational changes associated with the catalytic cycle presumably act to push LPS into the *β*-jellyroll domain of LptF or LptG, but more work is required to elucidate this mechanism [128].

**Figure 19:**
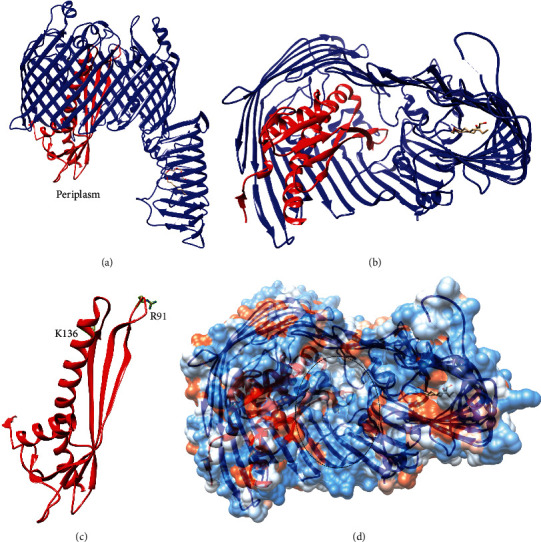
LptDE. (a) Ribbon structure of LptDE complex (PDB: 4Q35) [131]. LptD is colored blue, and LptE is colored red. A C8E detergent molecule is shown bound to the *N*-terminal *β*-jellyroll domain of LptD. (b) Periplasmic view of (a). (c) LptE from the LptDE complex in (a). Conserved basic residues shown to be important for LptE binding to LPS are highlighted [135]. (d) Hydrophobicity surface of (b). Hydrophobicity is shown in an orange-blue scale with orange as the hydrophobic end. The ellipse marks the periplasmic entrance to the LptDE lumen.

Finally, crystal structures of the LptDE outer membrane complex increase our understanding of how LPS is transported across the outer membrane and inserted into the extracellular leaflet [131, 136]. The structures reveal that LptD forms a 26-stranded *β*-barrel, and LptE forms a 4-stranded *β*-sheet and 2 *α*-helices (Figure 19) [131, 135, 136]. LptE acts as a plug for this *β*-barrel, and the extracellular side of the *β*-barrel is occluded by LptE and the extracellular loops of LptD [131, 136]. On the contrary, the periplasmic side of the *β*-barrel is open to reveal a large, hydrophilic lumen that may accommodate the polysaccharide of LPS (Figure 19) [131, 136]. LptE has been shown to bind LPS, and conserved basic residues of LptE that were found to be important for LPS binding are located at the extracellular side of the LptDE complex [128, 135, 136]. Therefore, movement of LptE upon LPS binding may allow these residues to interact with the polysaccharide, and this interaction with LptE may help feed the polysaccharide through the *β*-barrel [131, 135, 136]. The LptDE structures also revealed that the interactions of the first and final strands in the *β*-barrel are a weak point in the barrel structure thus suggesting that these strands may separate to allow the lipid A moiety of LPS to enter the membrane [131, 136]. As mentioned above, LptD also has an *N*-terminal, periplasmic *β*-jellyroll domain that likely forms the final subunit in the hydrophobic slide, and this domain was crystallized with a detergent molecule bound in its hydrophobic cleft (Figure 19) [127, 131]. This domain is located near the weak point in the *β*-barrel [131].

## 3. Conclusions

This review has summarized what is known about how LPS is synthesized and transported to the outer leaflet of the outer membrane with an emphasis on the structural and biochemical characterization of conserved proteins involved in these processes. While biochemical and structural research have provided a relatively detailed understanding of some aspects of LPS assembly, such as the synthesis of lipid A and the transport of completed LPS from the periplasmic leaflet of the inner membrane to the outer leaflet of the outer membrane, other aspects remain obscure, such as the transport and polymerization of O-antigen repeat units in the Wzy dependent pathway. Further biochemical and structural studies, particularly to obtain structures of Wzx and WaaL, will be required to complete our understanding of LPS assembly.

As discussed in Introduction, understanding the assembly of LPS has important implications for human health. The importance for LPS in bacterial viability and virulence makes the enzymes involved in this process promising targets for antibiotics, and the structural characterization of highly conserved enzymes may facilitate the rational design of broad-spectrum antibiotics targeting this pathway. Moreover, specific modifications in LPS alter the immunological response to this glycolipid, which often plays an important role in infection; in addition to modulating the inflammatory response mediated by the MD2/TLR4 complex, specific modifications to LPS can alter bacterial susceptibility to immune offences such as cationic antimicrobial peptides and the complement system [5, 8, 16, 19, 137–148]. Targeting the enzymes that make these specific modifications could lead to the development of antibiotics that selectively target specific pathogens without damaging normal microbiota.
